# Ecological consequences of neonicotinoid mixtures in streams

**DOI:** 10.1126/sciadv.abj8182

**Published:** 2022-04-13

**Authors:** Travis S. Schmidt, Janet L. Miller, Barbara J. Mahler, Peter C. Van Metre, Lisa H. Nowell, Mark W. Sandstrom, Daren M. Carlisle, Patrick W. Moran, Paul M. Bradley

**Affiliations:** 1 Wyoming-Montana Water Science Center, U.S. Geological Survey, Helena, MT 59601, USA.; 2 National Operations Center, Bureau of Land Management, Denver, CO 80225, USA.; 3 Texas Water Science Center, U.S. Geological Survey, Austin, TX 78754, USA.; 4 California Water Science Center, U.S. Geological Survey, Sacramento, CA 95819, USA.; 5 National Water Quality Laboratory, U.S. Geological Survey, Denver, CO 80225, USA.; 6 Earth System Processes Division, U.S. Geological Survey, Lawrence, KS 66049, USA.; 7 Washington Water Science Center, U.S. Geological Survey, Tacoma, WA 98402, USA.; 8 South Atlantic Water Science Center, U.S. Geological Survey, Columbia, SC 29210, USA.

## Abstract

Neonicotinoid mixtures are common in streams worldwide, but corresponding ecological responses are poorly understood. We combined experimental and observational studies to narrow this knowledge gap. The mesocosm experiment determined that concentrations of the neonicotinoids imidacloprid and clothianidin (range of exposures, 0 to 11.9 μg/liter) above the hazard concentration for 5% of species (0.017 and 0.010 μg/liter, respectively) caused a loss in taxa abundance and richness, disrupted adult emergence, and altered trophodynamics, while mixtures of the two neonicotinoids caused dose-dependent synergistic effects. In 85 Coastal California streams, neonicotinoids were commonly detected [59% of samples (*n* = 340), 72% of streams], frequently occurred as mixtures (56% of streams), and potential toxicity was dominated by imidacloprid (maximum = 1.92 μg/liter) and clothianidin (maximum = 2.51 μg/liter). Ecological responses in the field were consistent with the synergistic effects observed in the mesocosm experiment, indicating that neonicotinoid mixtures pose greater than expected risks to stream health.

## Teaser

Neonicotinoid insecticides are commonly observed as mixtures that can synergistically degrade ecosystem health.

## INTRODUCTION

Neonicotinoids are the most widely applied insecticides globally (*1*, *2*). Favored for their systemic action, rapid uptake, distribution in plants, and relatively low toxicity to vertebrates, neonicotinoids are used in agricultural settings as sprays and seed coatings and in urban environments as garden and companion animal treatments (*2*). Of the seven commercially available neonicotinoid compounds, imidacloprid (IMI), clothianidin (CLO), and thiamethoxam are the most heavily applied (*2*). Neonicotinoids gained global market share over other pesticides because of changes in regulations, target pest resistance, and pesticide application technologies (i.e., seed coatings) (*1*–*3*). However, neonicotinoids are increasingly implicated in declines in nontarget organisms and associated ecological services in terrestrial (*4*–*6*) and aquatic ecosystems (*7*–*9*) and may present a risk to human health (*10*). The European Union banned the outdoor use of neonicotinoids in 2018 (*11*), Canada has restricted some uses (*12*), and the U.S. Environmental Protection Agency (EPA) has recently suggested that IMI, CLO, and thiamethoxam may cause harm to a wide number of threatened and protected species (*13*–*15*). However, application of neonicotinoids remains widespread globally (*1*, *2*).

Neonicotinoids have been reported in recent surveys of surface waters in North America, often at levels that exceed regulatory guidelines and typically as mixtures (*8*, *16*, *17*). Effects of neonicotinoid mixtures on stream ecosystems are poorly understood because of regulatory and laboratory testing limitations. Pesticide testing, registration, use, and regulation target individual active compounds (*18*, *19*), whereas environmental exposures are predominantly to pesticide mixtures (*19*, *20*). As a result, nonadditive mixture risks [i.e., other than concentration addition (CA)] are not well documented (*19*, *20*). Further, because single-species toxicity tests are the regulatory standard, toxicity databases are dominated by readily available laboratory-cultured taxa that may not be representative of the full range of sensitivities observed in endemic ecological communities. A few laboratory (beaker) studies have reported nonadditive, including synergistic (greater than additive), effects of chronic exposures to neonicotinoid mixtures on a single species (*21*–*23*). However, synergistic effects of pesticide mixtures are considered rare and limited to laboratory settings and greater than environmentally relevant concentrations (*24*, *25*). To our knowledge, no study has documented pesticide mixture synergistic effects on aquatic ecosystems under both laboratory and field conditions.

Combining mesocosm and field studies (*26*–*28*) strengthens cause and effect interpretations by linking laboratory effect estimates directly to in situ exposure (pesticide concentrations) conditions while controlling for confounding factors like other chemicals and habitat degradation. While laboratory experiments are very useful (*29*), they are vulnerable to experimental bias and lack ecological relevance if not linked directly to observations from natural ecosystems (*30*). Conversely, elucidating cause (chemical mixtures) and effect (change in ecological communities) relationships is challenging in field (observational) studies because of numerous observed (*31*, *32*) and unobserved (*33*) confounding environmental conditions.

We conducted a 30-day mesocosm experiment to test the effects on aquatic communities of unary and binary exposures to IMI and CLO—two of the most toxic neonicotinoids (*34*, *35*) observed to co-occur frequently in U.S. streams (*8*, *16*, *36*, *37*). Our aim was to identify whether neonicotinoid mixtures might propagate lethal and sublethal ecological effects (i.e., emergence, trophic cascades, and predator-prey interactions) that diverge from predictions based on respective individual neonicotinoid exposures. Mesocosm results were then compared to a field dataset to further inform the ecological importance of these effect relations in streams of Coastal California.

## RESULTS

### Mesocosm results

**Fig. 1.**
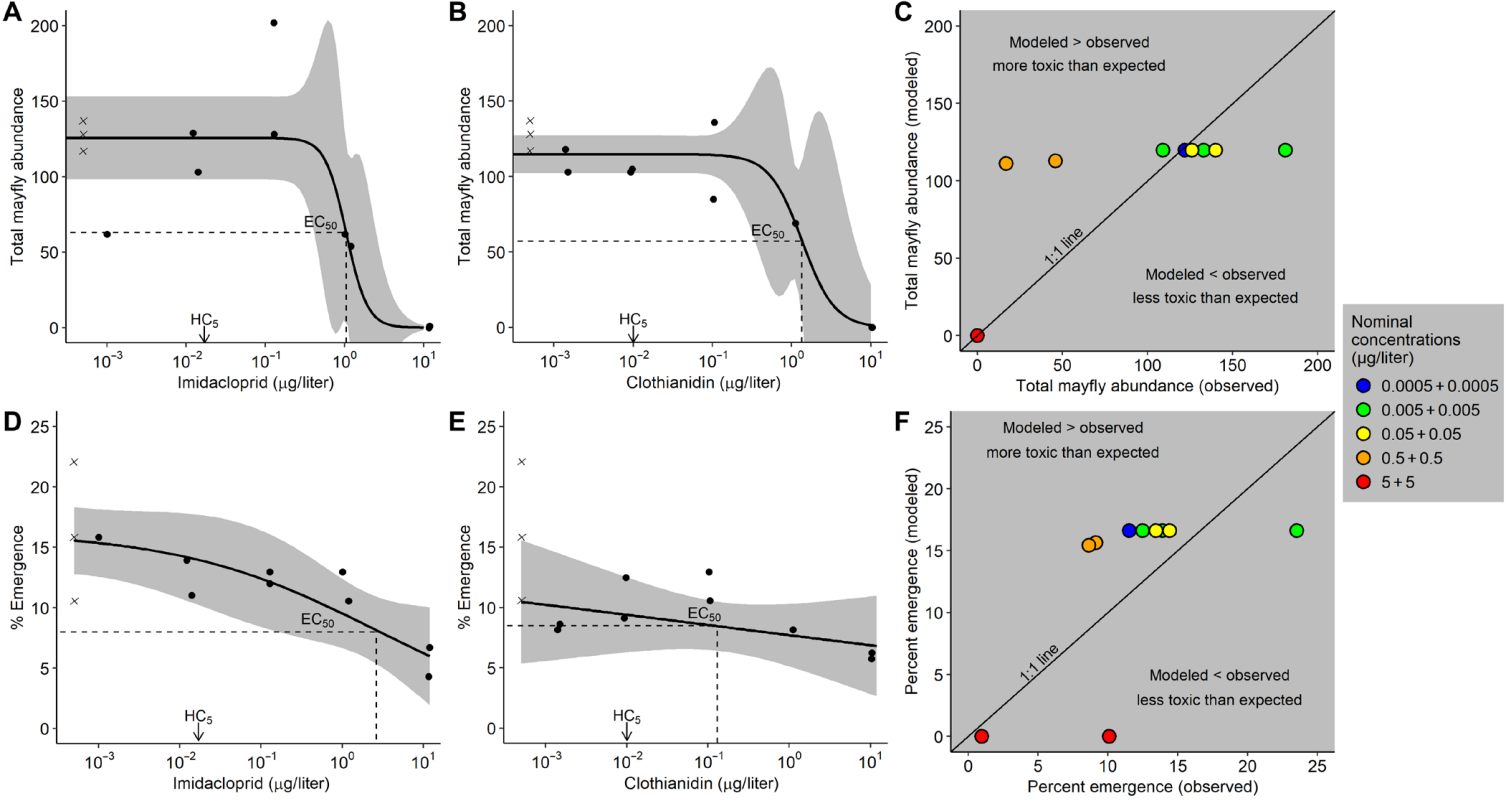
Total mayfly abundance, percent emergence, and response addition plots for IMI and CLO. For total mayfly abundance (**A** and **B**) and percent emergence (**D** and **E**), each data point represents an individual mesocosm stream, where circles are treatments and crosses are controls. In unary exposure models (A, B, D, and E), the *x* axis (log scale) is the time-weighted averages of measured concentrations (μg/liter), the black line is the three-parameter logistic regression line, and the gray ribbon is the 95% CI of the regression. In the response addition plots (**C** and **F**), observed results are for the binary exposures, and predicted values (modeled responses assuming additive effects) were based on the product of unary exposure response fits [see (A), (B), (D), and (E)]. Points above the 1:1 line (observed < modeled) in response addition plots (C and F) indicate a synergistic effect. EC_50_ is the 50% EC. HC_5_ is the hazard concentration at which 5% of species are affected derived from a species sensitivity distribution (see Fig. 4).

Taxa richness (range = 1 to 22 taxa), total abundance (range = 5 to 271 individuals per mesocosm or 120 to 6512 individuals/m^2^ basis for comparison with field data), total mayfly abundance (range = 0 to 202 individuals per mesocosm or 0 to 4854 individuals/m^2^), and other metrics of the benthic communities changed in response to exposure to neonicotinoids (table S1) (*36*). For the individual exposures (IMI or CLO), three-parameter logistic models were the best [based on the Akaike Information Criterion (AIC) (*38*)] model form across all taxa/taxa groups/community metrics examined except the mayfly *Ephemerella* spp. abundance for which the response to IMI was best fit using a four-parameter logistic model (Fig. 1, table S1, and figs. S3 to S17). Generally, unary exposure of IMI was less toxic to larvae and adult insects than unary exposures of CLO (table S1). For IMI, *Rhithrogena* spp. abundance was the most sensitive larval end point [based on a 20% effect concentration (EC_20_)], and scraper biomass was the least sensitive metric (table S1 and figs. S4 and S14). For CLO, *Ephemerella* spp. abundance was the most sensitive larval end point (EC_20_), and collector-filterer biomass was the least sensitive (table S1 and fig. S3 and S13). Total mayfly abundance, a community response metric, exhibited clear dose-response relationships to unary neonicotinoid exposures; 50% EC (EC_50_) values for IMI [EC_50_ = 1.05 ± 0.27 (SE) μg/liter] were nominally lower than for CLO (EC_50_ = 1.35 ± 0.52 μg/liter) (Fig. 1and table S1). For binary mixture exposures, the EC_50_ value (1.02 ± 0.09 μg/liter, reported as the sum of neonicotinoids in exposure) for total mayfly abundance was similar to the unary EC_50_ values for IMI and CLO (table S1). However, this was not the case for all taxa as 11 of the 17 mixture (1:1 mixture of IMI and CLO) EC_50_ values reported in table S1 were lower than the associated unary EC_50_ value for IMI or CLO. For example, the mixture EC_50_ value for total stonefly abundance was lower than the EC_50_ for IMI and CLO by a factor of >8 and 2, respectively. In four cases, the mixture EC_50_ was intermediate between the unary EC_50_ values for IMI and CLO, and in each of these cases, the EC_50_ value for CLO was substantially lower than the EC_50_ value for IMI. The mixture EC_50_ value was never higher than the unary EC_50_ value for IMI or CLO.

**Fig. 2.**
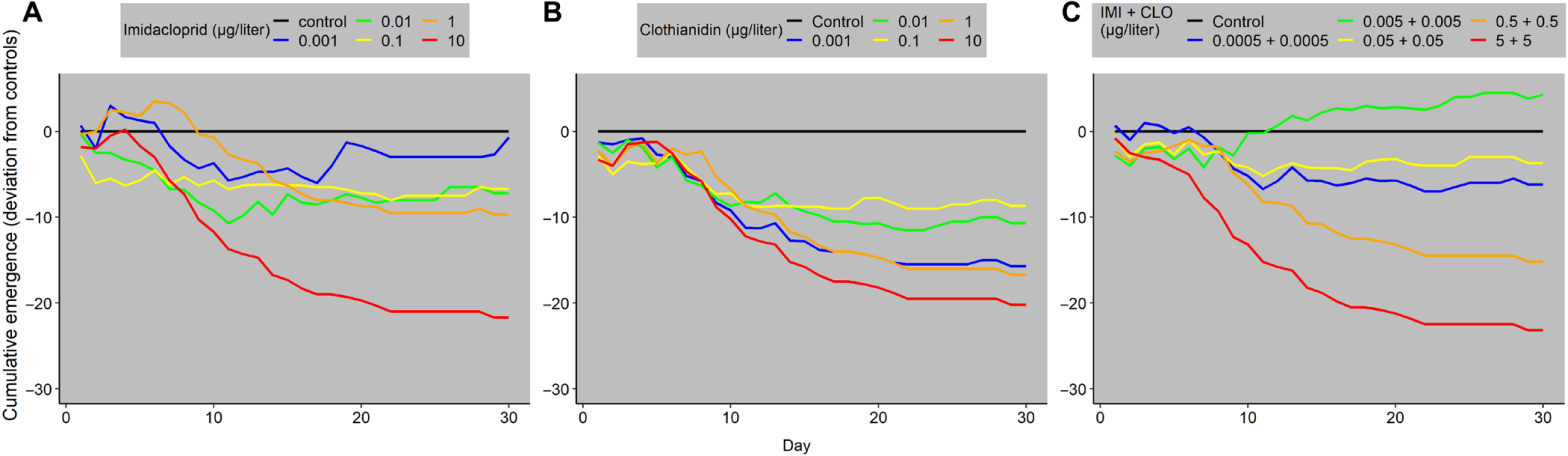
Average cumulative daily emergence minus average cumulative daily emergence from control streams. (**A**) IMI-only exposures, (**B**) CLO-only exposures, and (**C**) 1:1 mixture of IMI:CLO. *n* = 2 except for control (*n* = 3), 0.001 μg/liter for IMI (*n* = 1), 1 μg/liter for CLO (*n* = 1), mixture of 0.0005 + 0.0005 μg/liter (*n* = 1), and mixture of 0.005 + 0.005 μg/liter (*n* = 3). Concentrations reported are nominals. Error bars are not possible with this calculation, and no statistical test was performed.

Both unary and binary exposures of IMI and CLO altered the timing and total number of adult emergers, of which 83% were midges and blackflies and the remainder were mayflies. Some IMI treatments provoked initial stimulation of emergence, but general suppression of cumulative emergence was observed by day 30 (Fig. 2A). Across all CLO treatments, in contrast, cumulative emergence was suppressed relative to controls throughout the duration of the experiment (Fig. 2, A and B), but suppression did not increase with every increase in exposure. The binary exposures suppressed emergence across all doses (except for the 0.005 μg/liter treatment, Fig. 2C). As with the unary exposures to IMI and CLO, cumulative emergence was not suppressed with every increase in exposure (Fig. 2C).

Neonicotinoid mixtures caused synergistic toxicity effects (i.e., dose-dependent more-than-additive toxicity) to mayfly abundance (Fig. 1, C and F). The ratio of modeled/observed responses (model deviation ratio) for total mayfly abundance ranged from 0.66 to 6.55 along a gradient of low to high mixture concentrations. For low binary exposure concentrations (where the IMI + CLO concentration was less than the EC_50_ for the individual unary exposures), the observed total mayfly abundance was slightly higher (i.e., less toxic) than predicted (modeled) based on additivity. At binary mixture concentrations (IMI + CLO) comparable to the individual neonicotinoid EC_50_ values, however, total mayfly abundances were as much as a factor of 6.55 lower than expected for an additive response (except the 5 IMI + 5 CLO μg/liter treatment), indicating synergy at these higher concentrations (Fig. 1C). Predictions at the highest treatment (5 IMI + 5 CLO μg/liter) reached a zero limit (no insects emerged); thus, no deviation from the 1:1 line could be distinguished. In terms of emergence, the model deviation ratio ranged from 0.71 to 1.79, where all but one replicate were greater than 1, indicating greater than additive responses in IMI + CLO mixtures, with more synergy at higher exposure concentrations; the highest treatment was the exception, where the modeled response again reached the zero limit (Fig. 1F).

**Fig. 3.**
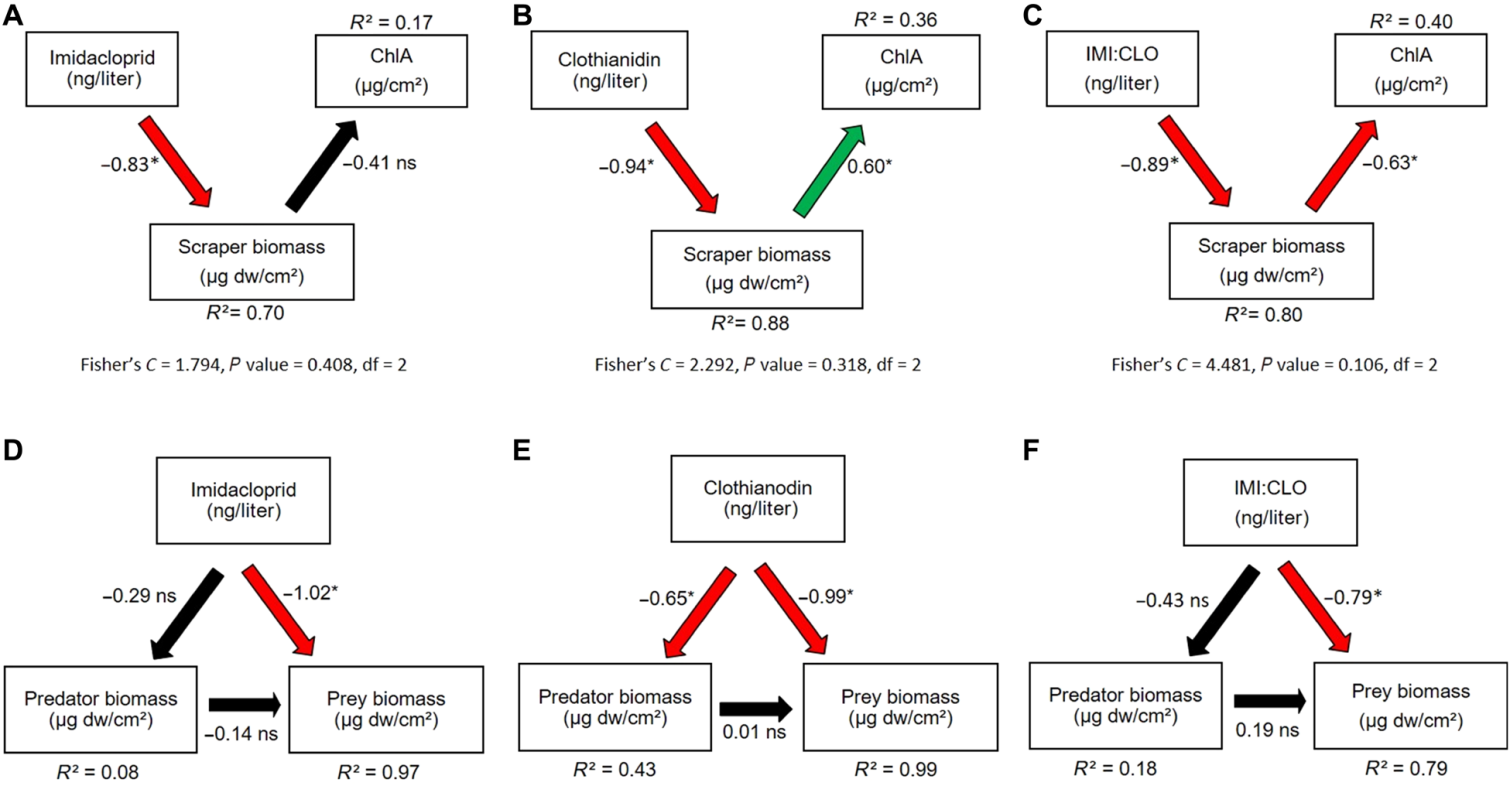
Path models depicting the direct and indirect effects of the neonicotinoids IMI and CLO on benthic community trophodynamics. Models (**A**) to (**C**) depict how neonicotinoid exposures affected scrapers and their consumption of algae (trophic cascade), and models (**D**) to (**F**) depict neonicotinoid effects on predator-prey interactions. IMI:CLO indicates exposure to a 1:1 mixture of IMI + CLO. Red arrows indicate statistically significant negative direct effects, green arrows indicate statistically significant positive direct effects, and black arrows indicate directed effects that were nonsignificant. Not depicted is the implicit assumption that IMI and CLO do not directly affect ChlA. Numbers are standardized path coefficients, and significance is designated by the following qualifiers: ns, not significant; *P < 0.05. A Fisher’s *C* value associated with a *P* > 0.05 indicated good model agreement and is only available for nonsaturated models (A to C).

Exposure to IMI and CLO substantially altered benthic community trophodynamics. Scraper biomass was directly and adversely (negatively) affected by unary exposure to IMI (standardized path coefficient = −0.83) and CLO (−0.94), with little evidence that either neonicotinoid caused indirect effects on chlorophyll *a* (ChlA) biomass (Fig. 3, A to C). While unary exposures to both neonicotinoids were directly toxic to scrapers, the relation between scraper and ChlA biomass was significant only in the CLO treatments and the relation was positive (Fig. 3, A and B, and fig. S18, A and B). These results do not support the hypothesis that a decrease in scraper biomass caused by exposure to an insecticide would cause an associated increase in algal biomass (i.e., a trophic cascade). As neonicotinoids are not expected to directly stimulate algal growth (no direct association was hypothesized in the model because there is no known mechanism), these findings for CLO are unexpected. In contrast, the binary neonicotinoid mixtures (Fig. 3C) caused a trophic cascade. Exposure to the binary mixture caused a direct decrease in scraper biomass (a direct effect, standardized coefficient = −0.89), while scraper biomass caused a direct decrease in ChlA (−0.63). The total effect of exposure to neonicotinoid mixtures caused the hypothesized increase in ChlA biomass (−0.89 multiplied by −0.63 = +0.56). The direct effect of the binary mixtures on scraper biomass was intermediate to those of the unary exposures to CLO and IMI. In the predator-prey models (Fig. 3, D to F), unary and binary exposures to neonicotinoids were directly toxic to prey, but only the unary CLO exposure was directly toxic to predators. The lack of a significant negative effect of predators on prey (an indirect effect of exposure to neonicotinoids) could indicate that exposure to neonicotinoids inhibited predator feeding (a sublethal effect) and decoupled predator-prey relations.

**Fig. 4.**
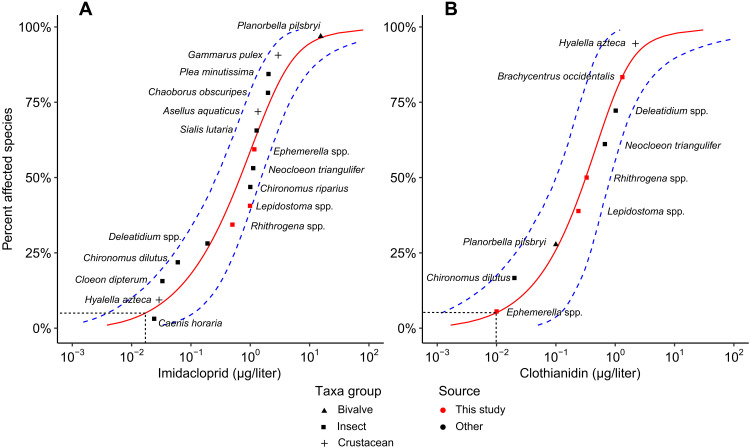
SSDs for aquatic invertebrates exposed to the neonicotinoids IMI and CLO. (**A**), IMI; (**B**), CLO. Data are EC_20_ values derived from the 30-day mesocosm test presented here (red symbols) and the lowest reported chronic (test duration, ≥17 days) effect value from the literature (black symbols). Marker shape indicates taxa group. Blue dashed lines indicate 95% CI. Horizontal dashed lines indicate the hazard concentration for 5% of the species (HC_5_): 0.017 μg/liter for IMI [± 0.030 SE, 95% CI (0.004 to 0.108)] and 0.010 μg/liter for CLO [± 0.034 SE, 95% CI (0.001 to 0.110)]. EPA aquatic life benchmarks for chronic toxicity to aquatic invertebrates are 0.01 μg/liter for IMI and 0.05 μg/liter for CLO.

Species sensitivity distributions (SSDs) and hazard concentration at which 5% of species are affected (HC_5_) values for IMI and CLO were developed from 25 taxa responses (16 for IMI and 9 for CLO) compiled from nine studies, including this study (Fig. 4). The IMI SSD (*n* = 16) included three taxa responses derived in this study, which fell within the range of previously published sensitivities (Fig. 4A). The CLO SSD (*n* = 9) included five taxa responses from the literature and was augmented with four novel taxa responses derived in this study, three of which were among the five most sensitive taxa responses reported for CLO (Fig. 4B). The resulting mean HC_5_ values were 0.017 μg/liter for IMI, which is comparable to the EPA chronic invertebrate aquatic life benchmark of 0.010 μg/liter, and HC_5_ = 0.010 μg/liter for CLO, which is five times lower than the current chronic invertebrate aquatic life benchmark of 0.05 μg/liter.

### Field results

At least one pesticide was detected in 86% of samples (*n* = 340) and at 97% of locations (*n* = 85) sampled in Coastal California streams. Total pesticide concentrations (sum of all pesticides, measured in nanogram per liter, reported in microgram per liter, and rounded to three significant digits) ranged from below the laboratory reporting level (reporting levels) to 115 μg/liter (Table 1). At least one EPA benchmark was exceeded at 40% of sample locations, with arithmetic mean chronic invertebrate benchmark quotient (CIBQ_total_, sum of benchmark quotients for all pesticides in the mixture; see Materials and Methods for details) = 7.47 (median = 0.41). At least one neonicotinoid was detected in 59% of samples and at 72% of locations sampled, and concentrations ranged from below laboratory reporting levels to 5.76 μg/liter (sum of all neonicotinoid concentrations). The arithmetic mean CIBQ_neonicotinods_ was 7.03 (median = 0.04) or 94% of the arithmetic mean CIBQ_total_. CIBQ_neonicotinods_ was greater than one in 28% of sampled locations. While dinotefuran was the most frequently detected neonicotinoid (61% of locations sampled), and thiamethoxam was observed to have the highest concentration (4.05 μg/liter), both IMI (42%) and CLO (45%) were frequently detected and observed at some of the highest concentrations (1.92 and 2.51 μg/liter for IMI and CLO, respectively) of any neonicotinoid detected. Two or more neonicotinoids (neonicotinoid mixtures) were detected at 56% of locations sampled, and at these sites, on average (arithmetic mean), CIBQ_neonicotinods_ accounted for 86% of CIBQ_total_ (*36*). IMI, CLO, or both were detected in a sample at 60% of sampled locations and co-occurred in a sample at 27% of sampled locations. Maximum concentrations of IMI and CLO were highly correlated (Pearson’s coefficient = 0.71), comprised, on average (arithmetic mean), 49% of CIBQ_total_ across all sites, while at sites with CIBQ_total_ > 0.1, IMI and CLO accounted for 67% of CIBQ_total_ (fig. S19) (*36*).

**Table 1.** Characteristics of neonicotinoid mixtures across the last 4 weeks of sampling of 85 wadeable streams (*n* = 340 samples) in Coastal California. <RL, less than the laboratory reporting level.

	**Detection frequency** (sample)*	**Detection frequency** **(site)^†^**	**50th, 75th percentiles,** **maximum concentrations** **(μg/liter) (sample)**	**Percent of sites > EPA** **benchmark^‡,§^ (site)**	**Mean^||^ (maximum** **concentrations/EPA** **benchmark^‡^) (site)**	**Mean^||^ (maximum** **concentration/** **HC_5_^¶^) (site)**
Sum of all pesticides	86%	97%	0.017, 0.076, 115	40%	7.47	10.91
Sum of all neonicotinoids	59%	72%	0.001, 0.010, 5.76	28%^#^	7.03^**^	10.46^††^
Acetamiprid	4%	5%	<RL, <RL, 0.29	0%	<0.01	
Clothianidin	30%	45%	<RL, 0.001, 2.51	6%	1.43	7.16
Dinotefuran	47%	61%	<RL, 0.002, 0.085	0%	<0.01	
Imidacloprid	28%	42%	<RL, 0.005, 1.92	25%	5.57	3.27
Sulfoxaflor	<1%	2%	<RL, <RL, 0.035	0%	<0.01	
Thiacloprid	0%	0%	<RL	0%	0.00	
Thiamethoxam	25%	32%	<RL, 0.001, 4.05	1%	0.03	

*Percentage of samples in which a neonicotinoid compound was detected during the last 4 weeks of sampling.

†Percentage of sites at which a neonicotinoid compound was detected during the last 4 weeks of sampling.

‡U.S. Environmental Protection Agency (EPA) chronic invertebrate benchmark.

**§**Percentage of sites where the 4-week maximum concentration exceeded the U.S. EPA chronic invertebrate benchmark.

||Arithmetic mean.

¶HC_5_ is the hazard concentration for fifth percentile of affected species detected in each statistic.

#Percentage of sites where the CIBQ for neonicotinoids was calculated by dividing the measured concentration for every neonicotinoid detected in a sample by its chronic invertebrate benchmark [usually the no observable adverse effect concentration (*34*)], summing them for each sample, and taking the maximum value of the four samples at each site.

**Mean of the 85 CIBQ_neonicotinoids_ and arithmetic mean of the 85 maximum toxic unit values (sum of seven neonicotinoid toxic units based on EPA benchmarks).

††Arithmetic mean of the 85 CIBQ_neonicotinoids_ values (replacing EPA benchmark values for IMI and CLO with the mesocosm derived HC_5_ values).

**Fig. 5.**
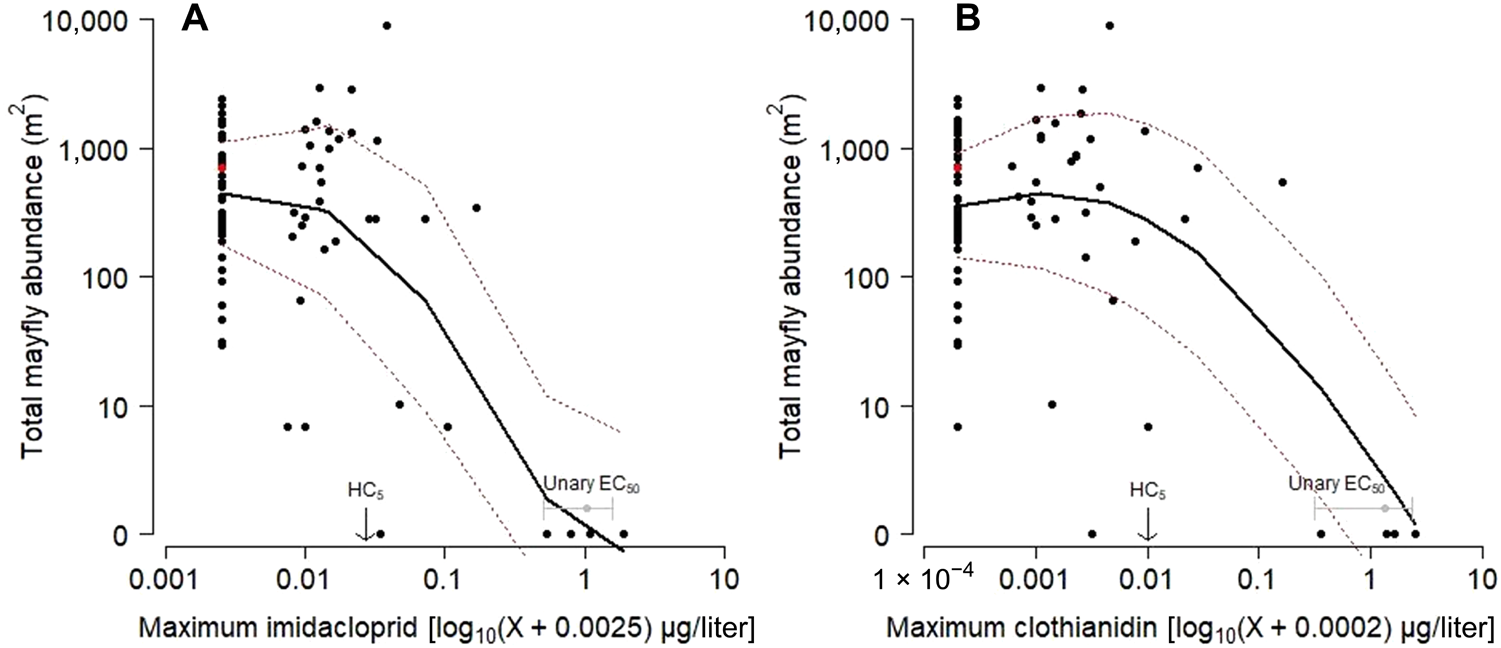
Total mayfly abundance [log(X + 1)] relations to neonicotinoid concentrations and metrics of pesticide toxicity [also log_10_(X + 1/2 lowest reported value)] observed in (*n* = 81) Coastal California streams (*52*). Note that the *x* axis in these plots are maximum concentrations of IMI or CLO; however, the model depicted is multivariate in nature. The generalized additive models depicted include the following covariates: Site elevation (recorded in meters), average soil bulk density (average across the watershed), and summer average precipitation (June to September 1971–2000 monthly average precipitation across the watershed in millimeters plus the interactive effect between CLO and IMI). Maximum concentrations were derived from the four weekly water samples collected immediately before the ecological survey at each site. The red dot indicates a sample removed from the analysis because it was determined to be a multivariate outlier during covariate analysis. EC_50_ values (bars are 2 SEs) were observed in unary exposures from the mesocosm experiment (see Fig. 1).

Total taxa richness in Coastal California (*n* = 82) streams ranged from 14 to 60 taxa, total taxa abundance ranged from 143 to 22,996 individuals/m^2^, and total mayfly abundance ranged from 0 to 8904 individuals/m^2^. Invertebrates were not collected at three sites (CA_DryMouth, CA_MArkWMir, and CA_Petaluma; table S7) where pesticides were collected and reported. One sample location (CA_Salinas; table S7) was determined to be a multivariate outlier (Mahalanobis D^2^) because of the natural covariates, resulting in 81 sample locations with paired pesticide and ecological data used in the generalized additive model (GAM) development. Site elevation (m), soil bulk density (averaged across the watershed), and summer average precipitation were significant natural covariates that described 47% of the variation (*R*^2^) of total mayfly abundance in Coastal California streams (table S8). All metrics of pesticide toxicity improved (AIC < 167.6) prediction of total mayfly abundance above that described by natural covariates (covariate only model AIC = 167.6; table S8). The top two models (lowest AIC scores) contained an IMI-CLO interaction term, and both models outperformed the model containing the CIBQ_total_ for all pesticides. The top model, which contained an IMI-CLO maximum concentration interaction term, described 72% of the variation in total mayfly abundance, displayed a dose-response shape consistent with that observed in the mesocosm experiment (Figs. 1 and 5, A and B), and had a break point near the mesocosm HC_5_. The field-derived total mayfly dose-response curves show precipitous declines in total mayfly abundance and local extirpation (zero mayflies observed at a site) when exposed to mixtures of IMI and CLO. Extirpation of mayflies in the field occurred at concentrations of IMI or CLO that only caused a 50% decline (EC_50_ for unary exposures) in total mayfly abundance when exposed to either compound alone (Fig. 5 and table S1).

## DISCUSSION

Mixtures of neonicotinoid compounds are commonly observed in stream ecosystems globally. We aimed to better understand the consequences of these mixtures to stream ecosystems by combining a 30-day mesocosm experiment with a field observational study. The mesocosm study determined that exposure to either IMI or CLO caused losses in stream invertebrate abundance, biomass, richness, and emergence, while exposure to mixtures of the two neonicotinoids disrupted trophodynamics and caused dose-dependent nonadditive (synergistic) effects in benthic communities. The field study evaluated the exposures to 253 pesticides, including seven neonicotinoids, in 85 small Coastal California streams and their relation to ecological health. Pesticide mixtures in Coastal California streams were complex, but potential toxicity (CIBQ_total_) was dominated by just two compounds: IMI and CLO. Mixtures of IMI and CLO were negatively associated with more than additive effects on ecological health. These patterns observed in the laboratory and field are consistent with the hypothesis that mixtures of IMI and CLO cause synergistic risks to aquatic life.

The mesocosm experiment improved estimates of toxicity thresholds of CLO for stream ecological communities. The unary treatments for CLO produced four new taxa responses including three of the most sensitive five taxa included in the chronic SSD (*Ephemerella* spp., *Rhithrogena* spp., and *Lepidostoma* spp., all mayflies) (Fig. 4B). We highlight that in the CLO SSD, the crustacean *Hyalella azteca* (widely considered to be a sensitive taxon to neonicotinoids) was 200 times less sensitive than the most sensitive aquatic insect (*Ephemerella* spp.). Once we augmented the dataset with these new species responses, the CLO SSD produced an HC_5_ value that is substantially lower than other CLO thresholds reported in the literature (*22*, *34*, *39*–*42*). A similar situation was recently reported for fipronil compounds, for which SSDs with limited aquatic insect taxa and corresponding data also resulted in underprotective toxicity thresholds for aquatic life (*28*). Given the dominance of noninsect taxa in toxicity databases (*43*), it is likely that noninsect taxa might drive toxicity estimates, potentially causing underprotective thresholds for other insecticide contaminants. In contrast, unary exposures for IMI produced results consistent with previous microcosm studies (*44*, *45*), an observational study (*9*), and the current EPA aquatic life benchmark (*34*). The IMI dataset of effect estimates before this investigation was robust, and the new taxa responses we added, while supportive of existing data, were not the most sensitive (Fig. 4A). These refinements in effect levels indicate that CLO is comparably or more toxic than IMI, an observation reported by others (*21*, *22*, *39*) and consistent with the fact that CLO binds more readily than IMI with the site of toxic action in aquatic insects (*46*).

Exposure to mixtures of neonicotinoids in the mesocosm experiment caused dose-dependent nonadditive (synergistic) effects to aquatic invertebrate communities. Exposures to mixtures of IMI and CLO with a sum concentration less than the individual neonicotinoid EC_50_ values caused additive or slightly less-than-additive effects on total mayfly abundance (Fig. 1C), but exposures with mixture concentrations exceeding the individual EC_50_ values for IMI and CLO caused effects that were substantially greater (model deviation ratio > 2, synergistic) than would be predicted from the assumption of additive effects (*25*, *47*). Greater than additive effects on percent emergence were observed in nearly every mixture treatment (Fig. 1F); however, deviations from expectation were never as severe (model deviation ratio < 2) as was observed for larval mortality (model deviation ration > 2). These findings are consistent with other laboratory studies that indicate that neonicotinoid mixtures caused dose-dependent synergistic effects in the midge *Chironomus dilutus* (*21*, *22*).

Neonicotinoid mixtures also disrupted trophic dynamics in the mesocosm experiment. The IMI + CLO mixture produced a trophic cascade, resulting in an accrual of algal biomass (Fig. 3), which was not observed in the unary IMI and CLO exposures. Trophic dynamics between invertebrate predators and prey, however, were disrupted in both unary and binary exposures. The unary exposure scenarios had a stronger effect on prey biomass. A hypothetical increase in predatory control on prey biomass, under reduced prey conditions, was not apparent. However, the results presented here are consistent with bioassay, mesocosm, and field studies that show that neonicotinoid exposure can inhibit feeding behaviors in invertebrates (*48*, *49*). Other insecticides [bifenthrin and fipronil (parent and three degradates)] have induced strong changes to trophic dynamics (trophic cascades) in mesocosms (*27*, *28*). Thus, our findings, particularly the ecologically relevant mixture treatments, contribute to a growing body of scientific evidence that indicates that pesticides commonly detected and often co-occurring in U.S. streams can decouple ecological processes.

Exposure to neonicotinoids altered emergence (Figs. 1, D to F, and 2), the ecological process whereby freshwater ecosystems sustain insect populations and support riparian consumers (*50*). IMI and CLO delayed or suppressed emergence even at very low exposure concentrations (0.001 μg/liter) (Fig. 2). Other insecticides (e.g., bifenthrin and fipronil) also alter the timing and abundance of aquatic insect emergence (*27*, *28*). The EC_50_ for percent emergence in CLO-dosed streams was lower than those for the most sensitive larval end points, indicating that—in contrast to previous findings for other pesticides (*28*)—emergence may be a more sensitive end point than larval mortality for CLO. Given the importance of the adult life stage of aquatic insects as food subsidies for other aquatic species and terrestrial consumers (*50*), these results indicate that neonicotinoids in aquatic ecosystems could decrease the number of prey items available for consumers in adjacent terrestrial food webs. Our findings (altered emergence in neonicotinoid exposures of >5 ng/liter) are consistent with observations in Europe where declines in insectivorous birds have been observed in regions with dissolved IMI concentrations of >19.4 ng/liter (*51*).

Pesticides were frequently detected (86% of samples) and occurred ubiquitously (97% of sites) in Coastal California streams. While pesticide concentrations ranged from below the laboratory reporting level to as high (sum of all pesticides) as 115 μg/liter, our monitoring missed many daily peak exposure events (*35*). Of the greater than 120 pesticides detected in Coastal California streams (*52*), mixtures of six neonicotinoid compounds accounted for a large fraction of the total predicted pesticide toxicity (CIBQ_neonicotinoids_ comprised 94% of mean CBIQ_total_). Neonicotinoids were detected in 72% of sampled streams and frequently comprised mixtures of dinotefuran, IMI, CLO, and thiamethoxam. Although not the most frequently detected (as percent of samples) nor widely distributed neonicotinoids (as percent of sites), IMI and CLO mixtures were commonly observed (60% of streams sampled, 27% of samples) and characterized a large fraction of the total predicted pesticide toxicity (49% of CIBQ_total_) observed in Coastal California streams. Other pesticides [e.g., fipronil (*52*)] were observed in these streams, but their collective predicted effects were relatively small (table S8 and fig. S19) compared to IMI and CLO.

The effect of neonicotinoid mixtures on benthic communities in Coastal California streams was greater than expected based on additive models of risk. The best model predictive of IMI and CLO mixture effects on total mayfly abundance, a group of invertebrates known to be sensitive to neonicotinoids (*53*, *54*), included more than additive effects. Effects to total mayfly abundance observed in the field were greater than expected due to exposure to either compound (IMI or CLO) individually (compare regression lines with EC_50_ values in Fig. 5 and table S1) or that were predicted to occur because of mixtures (table S1, mixture EC_50_ value). This indicates that IMI and CLO mixtures in the field caused greater than additive or synergistic effects, even greater than that observed in the mesocosm experiment. While this effects model accounted for the influences of other limiting factors [i.e., co-occurring pesticides and natural characteristics of the landscape (*31*)], we did not exhaust all possible factors (e.g., habitat degradation and food limitation) (*25*, *32*) that can interact with pesticide mixtures to cause synergistic effects to aquatic organisms (*25*, *55*). Despite these caveats, our findings are consistent with another study where effects observed in a semifield experiment were greater than anticipated from a series of laboratory experiments (*56*). Collectively, the laboratory and field observations indicate that mixtures of IMI and CLO can cause synergistic effects to aquatic communities.

The extent to which pesticides affect aquatic ecosystems has been a growing concern globally (*57*, *58*). We highlight that few sites/samples (3 and 14%, respectively) in the Coastal California regions were pesticide-free and that most (60%) of the streams sampled in Coastal California did not exceed a pesticide aquatic life benchmark. Our analysis also showed that total mayfly abundance was little affected at a wide range of low pesticide concentrations (see nonnegative slopes in Fig. 5 and fig. S19). Collectively, while most streams in Coastal California are affected by pesticides, there is little support for the idea that aquatic insect communities in Coastal California are suffering a catastrophic loss at large spatial scales due to exposures to pesticides (*30*, *59*, *60*). While others have detected at longer time (decades) and spatial scales (continents) that pesticide use is related to declines in aquatic insect diversity (*57*), our results neither assessed diversity nor made observations at space/time scales appropriate to measure such effects. Rather, neonicotinoid mixtures do pose risks to stream communities within the Coastal California region, and because of the ubiquity of IMI and CLO use globally (*2*), IMI and CLO likely cause local impairment around the world, the consequences of which are poorly understood.

## MATERIALS AND METHODS

### Mesocosm methods

We designed a 30-day stream mesocosm experiment to quantify risks to aquatic life associated with exposure to the neonicotinoids IMI and CLO. The mesocosm experiment had two purposes. The first was to expand the number of EC estimates for representative aquatic macroinvertebrates, especially aquatic insects, to support development of neonicotinoid SSDs and associated thresholds (*28*, *43*). The second was to evaluate whether effects of IMI and CLO binary mixtures are comparable to those predicted by addition of the two compounds’ individual effects (response addition—a laboratory equivalent to CA) based on ecological effect end points other than mortality (e.g., changes in community richness and indirect effects). A 30-day exposure was chosen to capture the full or near-full life cycle of many aquatic invertebrate taxa and to expand on the relatively few existing investigations (*17*) of neonicotinoid chronic (>10 days) exposure effects to aquatic invertebrates. This exposure regime is consistent with observations that neonicotinoids are observed in U.S. streams year round (*61*), in some cases occurring multiple times a week (*35*).

The mesocosm experiment was run at the Aquatic Experimental Laboratory (AXL), U.S. Geological Survey (USGS), Fort Collins, CO. Details of the design and operation of AXL have been previously described (*27*, *62*). Briefly, the experiment consisted of 33 recirculating (18.9 liters of high-density polyethylene buckets with a 13-cm-tall standpipe to control potential water volume to 6.98 liters) semiclosed [limited influx of organic matter and drifting larvae, yet recruitment from eggs is suspected (*27*, *62*)] experimental streams (mesocosms) distributed within four temperature-controlled water baths (Frigid Unit Living Streams). Each mesocosm received four polyethylene trays filled with 5-cm gravel that were deployed into the minimally disturbed Cache La Poudre River, CO, where the trays were colonized with benthic communities. Rock trays displace approximately 4.4 liters of water, resulting in a final mesocosm volume of 2.2 liters. Water was continuously renewed (5.7 liters/day or >2 volume replacements daily) and recirculated by an impeller drive pump at 1325 liters/hour (370 cm/s). Influent water had a mean pH of 7.6, conductivity of 90 μs/cm, dissolved organic carbon of 3.51 mg/liter, nitrate of 0.39 mg/liter, phosphate of 0 mg/liter, alkalinity of 20 mg/liter, and hardness of 17 mg/liter. Water temperature was maintained at 15°C to match the temperature in the Cache La Poudre River at the time of rock tray collection, and mesocosms were illuminated on a 16-hour light/8-hour dark cycle. Stream water for the mesocosms was collected from the location where rock trays were harvested and was stored in four 1700-liter polyethylene Ace Roto-Mold tanks in the laboratory.

The experimental design consisted of 30 treatment streams and three control streams. The treatment streams comprised three exposure series: a series of IMI, a series of CLO, and a binary series (IMI + CLO), each exposed to the treatment continuously for 30 days. Each exposure series consisted of five duplicated (two mesocosms each) treatment levels. IMI and CLO exposures ranged from 0.001 to 10 μg/liter, and binary exposures ranged from 0.001 to 5.0 μg/liter (each compound) in a 1:1 ratio [e.g., the highest concentration was 5.0 μg/liter for IMI (259.68 g/mol) + 5.0 μg/liter for CLO (249.68 g/mol), not equimolar, but molecular weights are similar]. We chose environmentally relevant concentrations based on measured neonicotinoid concentrations in aquatic systems (maximum reported concentrations: IMI = 320 μg/liter and CLO = 55.7 μg/liter) (*16*, *17*). The treatment level ranges also encompassed the lowest reported ECs for aquatic invertebrates: IMI 28-day EC_50_ = 0.126 μg/liter (immobility) and CLO 28-day EC_50_ = 1.0 μg/liter (emergence) (*17*, *39*, *49*); results from a shorter-duration IMI-only experiment of a 10-day effect estimated as low as IMI = 0.06 μg/liter for the mayfly *Baetis tricaudatus* (*63*); and current aquatic life benchmarks: IMI = 0.010 μg/liter and CLO = 0.05 μg/liter (*34*). Concentrated stock solutions were prepared by dissolving 50 mg of IMI [Chem Service, Chemical Abstract Service (CAS): 138261-41-3] or CLO (Chem Service, CAS: 210880-92-5) (purities > 90%) in deionized (DI) water and diluting each solution to a volume of 500 ml. Five 10-fold serial dilutions of each stock solution were made using DI water and stored at 4°C in amber glass bottles in the dark. Appropriate volume aliquots of the solutions were used to spike river water (20 liters) to achieve twice the targeted exposure concentration because spiked river water (doses) comprise 50% of the continuously renewed river water delivered to the mesocosm. To limit photolysis, treatment water was maintained either in lidded stainless-steel dose containers or in polyethylene carboys covered by black plastic bags.

Conductivity, temperature, and pH were recorded, and a water sample for pesticide analysis was collected from one control and one replicate of each treatment on days 3, 7, 10, 17, and 24. Dissolved oxygen was measured continuously in a subset of streams with PME MiniDOT loggers. Samples for analysis of neonicotinoid concentrations were collected by filtering 10 ml of water into a 20-ml amber glass vial using a Whatman 0.7-μm glass microfiber filer (GF/F) syringe filter (*64*). Samples were stored in the dark at 4°C and shipped to the USGS National Water Quality Laboratory (NWQL) for analysis. Neonicotinoid compounds were determined in water samples by direct aqueous injection liquid chromatography–tandem mass spectrometry (LC-MS/MS) by a modification of a previously published method (*65*). The modification was the use of a more sensitive LC-MS/MS instrument (Agilent 6495) that allowed injection of less sample (20 μl) to minimize bias from sample matrix (refer to the Supplementary Materials for more details). Samples from the CLO treatments that were below instrument detection levels (IDLs) (*n* = 13) using direct injection were concentrated using solid-phase extraction and reanalyzed. IDLs were estimated as the lowest calibration standard that met qualitative identification criteria and were 0.007 μg/liter for IMI and 0.003 μg/liter for CLO. Quality control and assurance procedures are provided in the “Method for determination of neonicotinoid compounds in mesocosm samples” section in the Supplementary Materials and tables S2 to S5. Briefly, measured exposure concentrations generally confirmed target concentrations, and any deviations observed were smaller than the differences among treatments (fig. S2). ChlA was measured in triplicate in every mesocosm on days 10, 20, and 29 using a BenthoTorch (bbe Moldaenke GmbH) portable fluorometer that provides real-time qualitative and quantitative data in situ (*66*). Each day, emergent adult insects were collected from nets that covered the opening of each mesocosm by aspiration and frozen for later identification. At the end of the 30-day exposure, the mesocosms and their contents (four rock trays) were scrubbed, decanted, sieved to 500 μm, and the dislodged invertebrates were preserved in 80% ethanol. Taxonomic identification of larval and adult invertebrates was completed by Timberline Aquatics (Fort Collins, CO) to the lowest taxonomic level possible, typically species except for chironomids that were generally identified to subfamily. Unless defined otherwise here, structural and functional metrics are calculated as described in Merritt and Cummins (*67*). Sensitive mayfly abundance is defined as the sum of the abundances of the mayflies that have proven sensitive to contaminants (*Ephemerella* spp. + *Rhithrogena* spp. + *Drunella* spp. + *Epeorus* spp.) (*43*, *68*). To calculate biomass, length measurements of individual larvae were converted to mass based on known relations between length and mass for each taxon (*69*) and then assigned to their appropriate functional feeding group (collector-gatherer, collector-filterer, scraper, predator, and shredder) to determine total biomass for each functional feeding group.

### Field methods

In Coastal California (fig. S1), small (wadeable) streams were sampled weekly for pesticides, followed by an ecological survey (*70*). Briefly, 85 stream sites were identified to span a range of agricultural and urban land uses as well as other land uses (*70*–*74*). Pesticide monitoring was timed to coincide with low-flow conditions during the growing season—a period of high pesticide use—and when stream invertebrate communities are mature and predominantly in the larval life stage. During April to June of 2017, one water sample was collected weekly at each site (fig. S1) for a period of 6 weeks at each site. Because the Coastal California study was part of a larger stream monitoring program (fig. S1; data are found at https://webapps.usgs.gov/rsqa/) where pesticides were monitored over different durations, only the last four water samples collected at each site are considered here. A 4-week water quality index period was selected because it was long enough for chronic exposures to biota and short enough that ecological communities likely had not recovered from the exposures. This 4-week period is also concordant with the length of the mesocosm experiment (30 days). Water samples were filtered at the site using a 20-ml polypropylene syringe to push the sample through a 0.7-μm GF/F (*65*) and analyzed at the NWQL by direct aqueous injection LC-MS/MS (Agilent 6495 triple quadrupole) (*52*, *65*) for pesticides (253 parent and degradate compounds) including the neonicotinoids acetamiprid, CLO, dinotefuran, IMI, sulfoxaflor, thiacloprid, and thiamethoxam. This is the same method as was used for the mesocosm experiment. A more complete description of this method including quality assurance and control and data interpretation are presented elsewhere (*37*, *52*).

Invertebrate communities were sampled following the last pesticide sampling. Invertebrate community sampling was done at all 85 sites where pesticides were collected using a Surber sampler or D-frame net with 500-μm mesh openings. All invertebrates were identified and enumerated to either genus or species level at the NWQL (*75*). All chemical and biological data collected in the field and used in this manuscript are provided in the companion data releases available at https://doi.org/10.5066/P9D958A0 and https://doi.org/10.5066/P9ADZYQE (*36*, *37*).

### Mesocosm data analysis

Dose-response curves were fitted to time-weighted exposure concentrations (see the Supplementary Materials for details) and to responses (i.e., larvae abundance, composite metrics, and total adult insect emergence; see table S1) using logistic models in R (version 3.6.1) extension package “drc” (*76*). AIC (*38*) was used to select between three- and four-parameter logistic models (lowest AIC_score_ indicates the best model) for each model fit. When confidence intervals (CIs) of the regression at the intercept did not include zero, ECs (EC_20_ and EC_50_) were calculated, and model fit was evaluated using the Nash-Sutcliff coefficient (*45*). Otherwise, models were not fit to the data, and no ECs were reported.

To assess effects on emergence timing, the cumulative daily emergence (count of all individuals) of insects from mesocosms normalized to controls was computed by subtracting the mean emergence in the control mesocosms from the emergence in each treatment mesocosm. Effects on total emergence were assessed as the percent total emergence from each stream mesocosm, computed as the ratio of the number of emerged adult insects from a given mesocosm to the mean sum of larvae + adults in controls.

To evaluate the additive effects of neonicotinoid mixtures, response addition was determined separately for larvae and adults. First, a three-parameter logistic equation was fit to the time-weighted average (mean) of IMI or CLO and either total abundance of mayfly larvae or percent total emergence (all adults not just mayflies) (*28*). The individual contaminant fits were used to predict the response to the binary mixtures (IMI + CLO) at each mixture exposure, assuming response addition (*68*), and the modeled response versus observed total mayflies or percent emergence was plotted. Last, we calculated the model deviation ratio (model deviation ratio > 2 indicates synergism) (*25*, *47*) by dividing the modeled response by the observed response for each replicate of the mixture treatments.

To test how trophodynamics of benthic communities were affected by exposure to unary and binary mixtures of neonicotinoids and controls, we tested simple networks of cause-effect relationships describing a trophic cascade and predator-prey relationships using a path-analytic approach (R package “piecewiseSEM”) (*46*). The presence of IMI or CLO individually and as a mixture was hypothesized to directly reduce the biomass of scrapers and to indirectly cause an increase in the biomass of ChlA due to reduced grazing pressure (*47*). We also evaluated the effects of IMI and CLO individually and as a mixture on predators and prey (all nonpredatory insects) that indirectly affected predatory control of prey density. Compound concentrations were the predictor variables, and ChlA and the biomass of insects with the ecological feeding traits scraper, predator, and nonpredators (prey) were response variables. The Fisher’s *C* statistic was used to assess the general fit of each model, where *P* values of <0.05 were defined as indicating a good fit (*46*) for the trophic cascade models; because the predator-prey models are saturated, there are no degrees of freedom to test the general model fit. Models were not contrasted against one another.

We estimated the concentration at which only 5% of species are affected, i.e., protective of 95% of species, a risk-based hazard concentration (HC_5_). To do so, we used the EPA ECOTOX database (https://cfpub.epa.gov/ecotox/, accessed 14 March 2019) and values from the literature (*17*, *22*, *36*, *39*–*42*, *54*, *77*–*79*) to obtain ECs for IMI and CLO. These data were filtered to include only chronic exposures (≥10 days) for aquatic insects and crustacea and were combined with the chronic (30-day) taxa-specific toxicity data generated from our mesocosm study. For any given effect estimate (lethal or nonlethal), the lowest reported effect value for any taxa response was retained and the lowest effect estimates were included in the SSD dataset. We then used the R package “ssdtools” version 0.3.4 (14 May 2021) to estimate HC_5_ values [weighted average from values estimated from the following four distributions (log-normal, log-gumbel, gamma, and Weibull)] and 95% confidence intervals (*80*).

### Field data analysis

We assessed the detection frequency of any parent compound (degradates excluded) detected in streams in the Coastal California region on a per sample and per site basis using the last four weekly samples collected during the sampling campaign. Only detections above the laboratory reporting levels, including some estimated concentrations (estimated because recoveries exceeded 100% ± 30%) of parent compounds, were used in calculating detection frequencies, values below reporting levels were censored (set to zero for detection frequencies) and other summary statistics (50th, 95th, maximum concentrations) (*37*).

Predictors of pesticide toxicity evaluated included metrics that assumed additive toxicity of mixtures including a measure of total pesticide stress, the CIBQ on a per site basis. CIBQ is calculated by dividing the measured concentration for every pesticide detected in a sample by its chronic invertebrate benchmark [usually the no observable adverse effect concentration (*34*)], summing them for each sample, and taking the maximum value of the four samples observed at each site. Potential toxicity of all pesticides (CIBQ_total_), all neonicotinoids (CIBQ_neonicotinoids_), and for each of the neonicotinoid compounds were calculated separately as the arithmetic mean for all sampled streams (*34*). The EPA chronic aquatic life benchmark quotients for each of the neonicotinoids are as follows: acetamiprid (2.1 μg/liter), CLO (0.05 μg/liter), dinotefuran (> 95,300 μg/liter), IMI (0.01 μg/liter), sulfoxaflor (>50,500 μg/liter), thiacloprid (0.97 μg/liter), and thiamethoxam (0.74 μg/liter). The frequency of exceedances (CIBQ > 1) was also evaluated for all pesticides (CIBQ_total_) and all neonicotinoids (CIBQ_neonicotinoids_), and each neonicotinoid compound were calculated separately. For comparison, we also computed the arithmetic mean CIBQ_total_, CIBQ_neonicotinoids_, and for IMI and CLO by replacing the chronic invertebrate benchmark for IMI and CLO with the mesocosm-derived HC_5_ values.

Relations between aquatic life and exposure to pesticides were explored via GAMs (“mgcv” package in R (*52*)). GAMs relate a univariate response variable (i.e., total mayfly abundance) with parametric predictors (i.e., measured of pesticide exposure and covariates) that depend on nonparametric smoother functions. First, we related total mayfly abundance to 10 natural covariates used in California Stream Condition Index to account for naturally occurring variation in aquatic assemblages and to minimize confounding (spurious associations between natural and anthropogenic factors) with anthropogenic stressors, in this case, pesticides (*81*, *82*). The covariates represent measures of site location (latitude, longitude, site elevation, and elevation range of watershed), basin size, long-term air temperature, precipitation (long-term and summer average), bulk soil density, and soil erodibility (*81*). All 10 natural covariates were included in the initial model and sequentially removed until all the remaining covariates significantly contributed to the covariate only model. The top covariate model was selected on the basis of the lowest AIC value (*38*). Second, one of eight predictors of pesticide toxicity (table S8) was added to the covariate-only model to determine whether residual variation in total mayfly abundance was due to pesticide toxicity. The inclusion of a pesticide toxicity metric was evaluated to be important if the pesticide metric slope was statistically significant (*P* ≤ 0.05) and the resulting AIC value was less than the covariate only model (*38*). The model with the lowest AIC value was determined to be the best model. Predictors of pesticide toxicity included six indices that assume additive pesticide toxicity including CIBQ_total_, CIBQ_neonicotinoids_, CIBQ_IMI_, CIBQ_CLOm_, and the maximum observed concentration of IMI or CLO as individual predictors of total mayfly abundance. Last, to consider nonadditive toxicity, two interaction models were evaluated, the interaction between CIBQ_IMI_ × CIBQ_CLO_ and the interaction between the maximum observed concentration of IMI × maximum observed concentration of CLO. Total mayfly abundance was log_10_(X + 1)–transformed, whereas pesticide metrics were transformed using log_10_[X + ½ the lowest reported value (IMI = 0.005 and CLO = 0.0004)]. The use of invertebrate metrics total mayfly abundance facilitates the direct comparison of the dose responses observed in the mesocosm study with those observed in the field study.
