# Supplementary material for: Ecological consequences of neonicotinoid mixtures in streams
Source: Sci Adv. 2022 Apr 13;8(15):eabj8182. doi: 10.1126/sciadv.abj8182 (PMC9007503; doi:10.1126/sciadv.abj8182)
Supplement: Supplementary file 1 — Supplementary Text Figs. S1 to S19 Tables S1 to S8 [file sciadv.abj8182_sm.pdf]

Science Advances
AAAS

Supplementary Materials for

Ecological consequences of neonicotinoid mixtures in streams

Travis S. Schmidt*, Janet L. Miller, Barbara J. Mahler, Peter C. Van Metre, Lisa H. Nowell, Mark W. Sandstrom, Daren M. Carlisle, Patrick W. Moran, Paul M. Bradley

*Corresponding author. Email: tschmidt@usgs.gov

Published 13 April 2022, *Sci. Adv.* **8**, eabj8182 (2022)
DOI: 10.1126/sciadv.abj8182

This PDF file includes:

Supplementary Text
Figs. S1 to S19
Tables S1 to S8

Supplementary Text

Data analysis

Dose-response

Effect concentrations at which a 20 or 50% change was observed (i.e., EC_{20}, EC_{50}) in larvae abundance when exposed to imidacloprid (IMI) and clothianidin (CLO) were calculated by fitting the data (x= time-weighted mean neonicotinoid concentration, y= larval abundance) using logistic models in R (version 3.6.1) (*1*) extension package "drc" (*2*). Exposure concentrations were characterized by time-weighted averages of measured concentrations, whereby each observation was weighted by the number of days between the start of the mesocosm experiment and the next observation, between observations, or between last observation and the end of the experiment. If a compound was not detected in a stream mesocosm and the nominal (treatment target concentration) concentration was >0, an estimated value of 0.5 times the instrument detection level (IDL) was substituted. Akaike Information Criteria (AIC) (*3*) were used to select between 3- and 4-parameter logistic models for each model fit. Curves were fit to all taxa with adequate abundance (where confidence intervals of regression at the intercept did not include zero) and to aggregate metrics (e.g., sensitive mayfly abundance) for the purpose of estimating effects on populations and communities. Model fit was assessed using the Nash-Sutcliff Coefficient (NSC) (*45*), where a poor model fit can receive infinitely negative values and a perfect fit receives a value of 1.

Method for Determination of Neonicotinoid Compounds in Mesocosm Samples

Neonicotinoid compounds were determined in water samples by direct aqueous injection (DAI) liquid chromatography tandem mass spectrometry (LC-MS/MS). The method was a modification of a previously published method for 225 pesticide compounds (*4*). The modification was the use of a more sensitive LC-MS/MS instrument (Agilent 6495) that allowed injection of less sample (20 µL) to minimize bias from sample matrix.

Water samples were measured with an Agilent 1290 Infinity LC system consisting of a binary pump, autosampler, sample tray cooler, and heated column compartment coupled to an Agilent 6495 triple quadrupole system equipped with an Agilent Jet Stream electrospray ionization source. The analytical column used for compound separation was a Zorbax C18 column (50 mm x 2.1 mm i.d., 3.5 µm particle size), maintained at 40 °C. The mobile phase consisted of 1 mM formic acid/water (phase A) and methanol (phase B). The gradient started with 100 percent phase A, changed to 80 percent at 4 min, 60 percent at 5 min, 40 percent at 8 min, 25 percent at 13 min, 10 percent at 15 min, and 100 percent at 17 min. The flow rate was 0.6 mL/min and the injection volume was 20 µL.

Tandem mass spectrometry was carried out using positive electrospray ionization and multiple reaction monitoring (MRM). The precursor and product ions and optimum collision energy were selected from analysis of each individual compound. The retention times and precursor and product ions for each compound are shown in Table S2. The precursor ion obtained was the molecular ion $[M+H]^+$ for all compounds except imidacloprid-olefin, which decomposed in the source to a fragment ion of 208.1 m/z. Qualitative criteria for identification of the compounds in a sample were that both MRM peaks overlapped and had retention times within 0.1 min of calibration standards, and that MRM ion ratios from samples were within

±30% (relative) of calibration standards.

The MRM ionization source parameters were optimized sequentially by injecting the pesticide compound mixture at a concentration of 10 ng/mL and selecting values that provided the largest response for most of the compounds. The optimized acquisition parameters were drying gas temperature 150 °C, drying gas flow 15 L/min, nebulizer 240 kPa, capillary voltage 2,000 V, nozzle voltage 0 V, sheath gas temperature 400 °C, sheath gas flow 12 L/min. The ion funnel parameters were high pressure RF voltage 90 V, and low-pressure RF voltage 80 V.

Samples were analyzed in a sequence of instrument blanks, calibration standards, laboratory quality control (QC) samples, and environmental samples; this sequence is called an analytical batch. The laboratory QC samples include blank samples and spike samples. Spike samples included preparation spike samples and instrument continuing calibration verification (CCV) samples (both at 250 ng/L), and instrument detection level (IDL) standards (5,10, 25 ng/L). Blank samples included preparation blank samples and instrument continuing calibration blank (CCB) samples. The laboratory preparation blanks and spikes were prepared in 20-mL sample containers similar to those used for the environmental samples, and instrument blank and spikes samples were prepared in 2-mL analytical vials. Low concentration standards were used as IDL standards to check LC-MS/MS response during and at the end of the analytical batch. A typical analytical batch consisted of about 109 analytical vials with 75 environmental samples, 2 preparation QC samples, and 32 instrument QC samples (blanks, calibration standards, CCVs).

The mesocosm stream samples were analyzed in two analytical batches. Data from each analytical batch was analyzed using the MassHunter quantitative analysis software. Calibration was performed using the peak areas and the internal standard technique. A series of 12 calibration standards, ranging from 1 to 10,000 ng/L, were analyzed at the start of each batch. Calibration curves were generated using the MassHunter software using quadratic curve fit, ignore origin, and 1/x weighting settings. The calibration curves used for the mesocosm sample met acceptance criteria of the fit of quadratic curve (r^2) greater than 0.990, and bias of each calibration standard in the curve relative to the nominal concentration less than ±30 percent, except at the lowest level where bias was ±50 percent (*4*).

The method detection limit (MDL) was determined from an estimate of the standard deviation of low-concentration samples based on the U.S. Environmental Protection Agency (EPA) test procedure guidelines for analysis of pollutants (40 CFR, part 136). The limits of quantitation (laboratory reporting level, LRLs) were initially calculated as twice the MDL (*5*). The calculated method detection levels were 7 ng/L for IMI and 3 ng/L for CLO. Samples with no MRM response or that failed qualitative identification criteria were reported as <LRL.

Isotopically labeled standards IMI-d_4, CLO-d_3, and atrazine-d_6 were used as internal standards for quantitation and were added to all laboratory quality-control samples and mesocosm stream samples. IMI-d_4 and CLO-d_3 were used as Internal Standards (ISTDs) for quantitation of the neonicotinoid compounds, and atrazine-d_6 was used for quantitation of the neonicotinoid ISTDs. Recoveries are shown in Table S3. Median recovery of IMI-d_4 was 102.5% (85.8 to 131%) and CLO-d_3 was 103.1% (83.8 to 127%); these generally were within the method

acceptance criteria of 70 to 130%.

There were 11 laboratory instrument blank and preparation blank samples analyzed with the mesocosm stream samples (Table S4). There were no detections of CLO or IMI in any of the blank samples, except for one CCB sample. The blank sample (CCB6) analyzed at the end of one batch had carryover from diluted nominal spike samples (100 mg/L at 50X dilution) analyzed in the sequence immediately before that and other QC samples.

Recoveries of neonicotinoid compounds in laboratory spike samples are shown in Table S5. One CCV sample (CCV6) and the lowest IDL samples (5 and 10 ng/L) analyzed at the end of one batch were excluded from this summary because of carryover from samples (mesocosm nominal stock solutions 100 mg/L diluted 50 times) analyzed in the sequence immediately before these samples. Median recovery of the neonicotinoids was 103.2% (80.1 to 130%) in all spike samples, except for IMI in the 5 ng/L IDL sample (recovery 38.29%), which was spiked below the MDL. These recoveries were within acceptance criteria used for the original method (70 to 130%).

Many of the samples from mesocosm experiments with nominal concentrations of IMI and CLO between 0.5 and 1 ng/L were reported as non-detections because the MDLs for these compounds were 3 and 7 ng/L. We processed a subset of the samples in 2019 using solid-phase extraction (SPE) to increase the sample mass for analysis. The purpose of the modification was to concentrate the pesticides so that they could be quantified at lower concentrations than possible with direct injection LC-MS/MS.

The procedure used to isolate pesticides from water was a modified version of the manual C-18 solid-phase extraction (SPE) method (*6*). The modification was to use a small volume (5–10 mL rather than 1,000 mL), manual sample pump rather than the AutoTrace automated system and to use different solvents for elution and keeper. The SPE columns were obtained from Biotage (Isolute C-18 (EC), 50 mg/3 mL, stainless steel frits (221-0005-BS). The SPE columns were eluted with 250 µL of hexane/acetone (1:1) and allowed to drain into a 15-mL culture tube containing 90 µL of methanol, followed by a second addition of the elution solvent. After elution, the elution solvent was evaporated with a stream of nitrogen using a TurboVap evaporative chamber set at a temperature of 30 °C and nitrogen pressure of 4 lb/in^2.

Recoveries of CLO in six spike samples at concentrations from 0.5 to 5 ng/L after SPE were from 91 to 147%. The SPE procedure for CLO provided an improvement in detection of low concentrations. Three additional detections were reported for the CLO treatment at 1 ng/L nominal concentration, and four additional detections were reported for the mixture treatment at 0.5 ng/L nominal concentration.

RESULTS

Water-quality and pesticide samples in mesocosms

Water-quality measurements were similar among mesocosms throughout the experiment with mean values (±standard deviation) for temperature, pH, and conductivity of 15 (± 0.6) ˚C, 7.6 (± 0.1), and 90 (± 3.6) µS/cm, respectively. Mean dissolved oxygen (DO) was >8.0 mg/L

(based on continuously monitored DO readings including day and night observations) and was on average 80% of saturation (elevation =1525 meters (World Geodetic System 1984)). Two replicates from the unary series (one each from the IMI and CLO exposures) were excluded from analysis because of experimental error resulting in n=31. For the remaining treatments, measured IMI and CLO concentrations approximated nominal concentrations (Fig. S2) with time-weighted average concentrations ranging from IDL to 11.9 µg/L for IMI and from IDL to 10.4 µg/L for CLO (*7*). All control treatments had IMI and CLO concentrations less than the limit of quantitation (<LRL). Early (days 3 and 7) measurements of IMI and CLO concentrations were generally lower than those measured later (days 17 and 24) in the study because of system design (4–10 days are necessary to achieve steady state under ideal conditions) (*8*). The one exception was that the target treatment (0.001 µg/L) for IMI had concentrations near 0.10 µg/L. Otherwise, variability in exposure over time was small and generally less than differences among treatments (Fig. S2). Three CLO-only streams had detectable levels of IMI, each of which had a time-weighted average of < 0.002 µg/L and occurred in CLO treatments > 0.01 µg/L. Similarly, three IMI-only streams had detectable CLO levels with time-weighted averages < 0.001 and occurred in IMI treatments > 0.01 IMI. Finally, we also detected imidacloprid-olefin in mesocosm samples. Imidacloprid-olefin is a degradate of IMI and was measured to be between 0.1 and 0.5% of the measured IMI concentrations, indicating little degradation of the parent compound. Because detected concentrations were substantially less than effect concentrations and there were no other reasons to distinguish these streams from uncontaminated replicates, the influence of these non-target compounds on results of treatment compounds was inferred to be minimal.

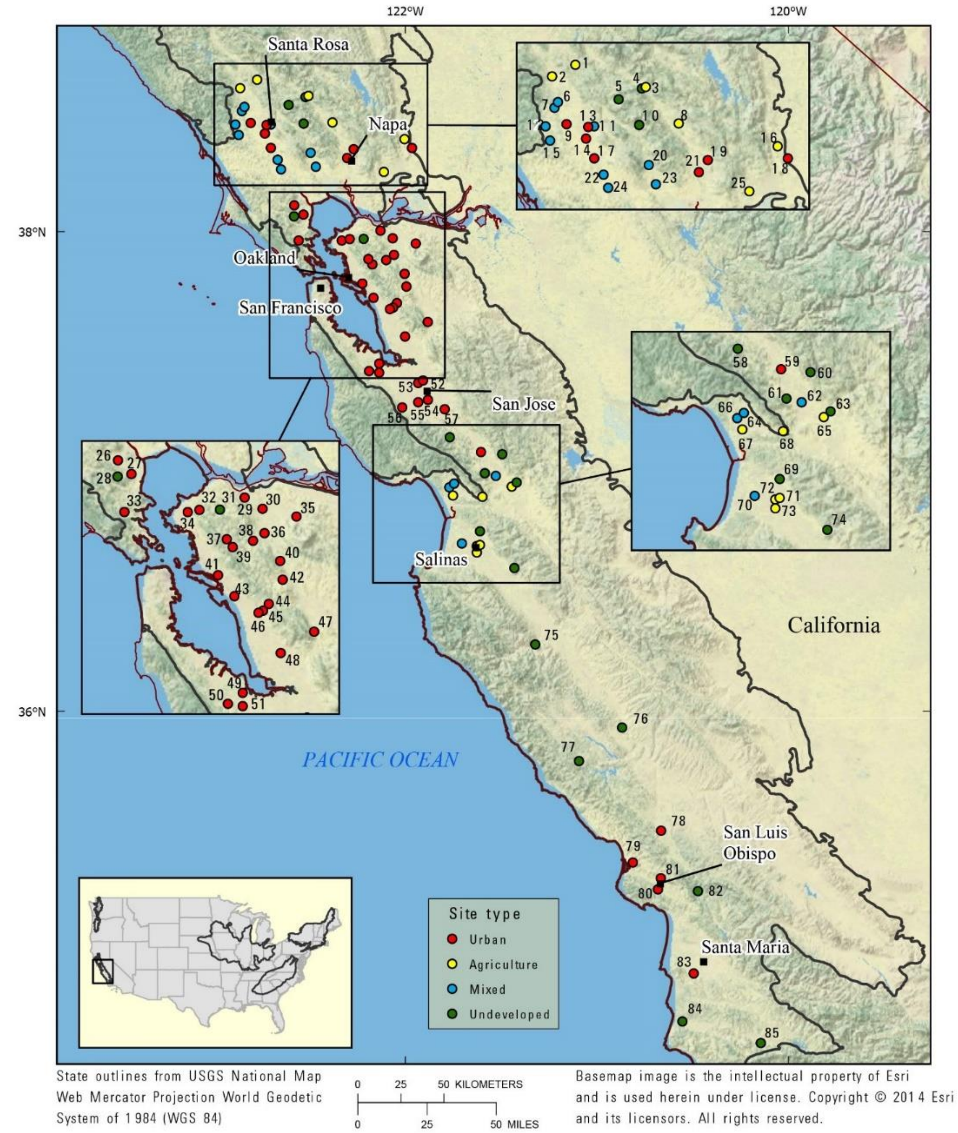

Fig. S1. Map of the Coastal California ecoregion sampled in 2017 as well as the other regions that were the subject of the Regional Stream Quality Assessement 2013-2017. Sites numbers described in table S7.

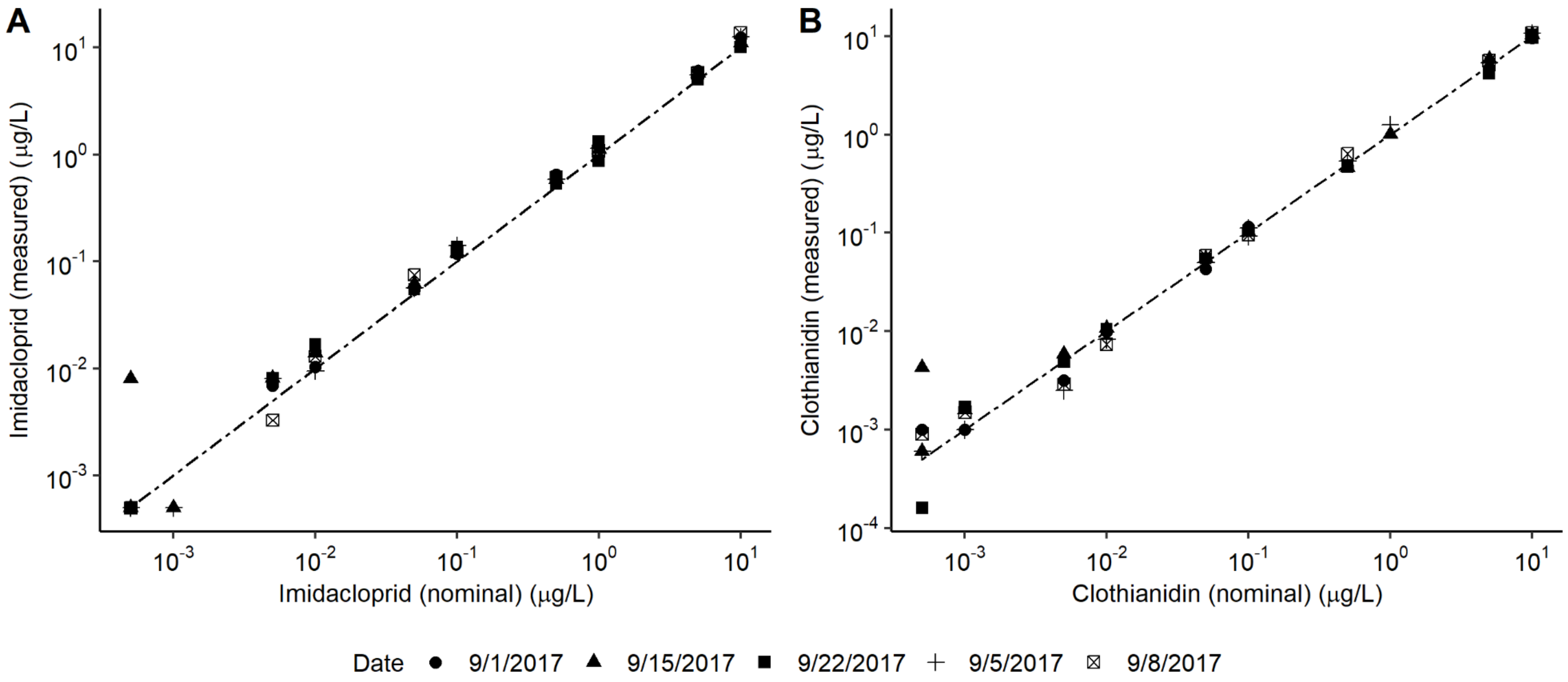

Fig. S2. Measured concentrations (μg/L) of (A) imidacloprid and (B) clothianidin plotted against nominal concentrations (μg/L). Axes are on log_{10} scale. Dashed line is the 1:1 line. Legend indicates the day of the experiment that the sample was collected.

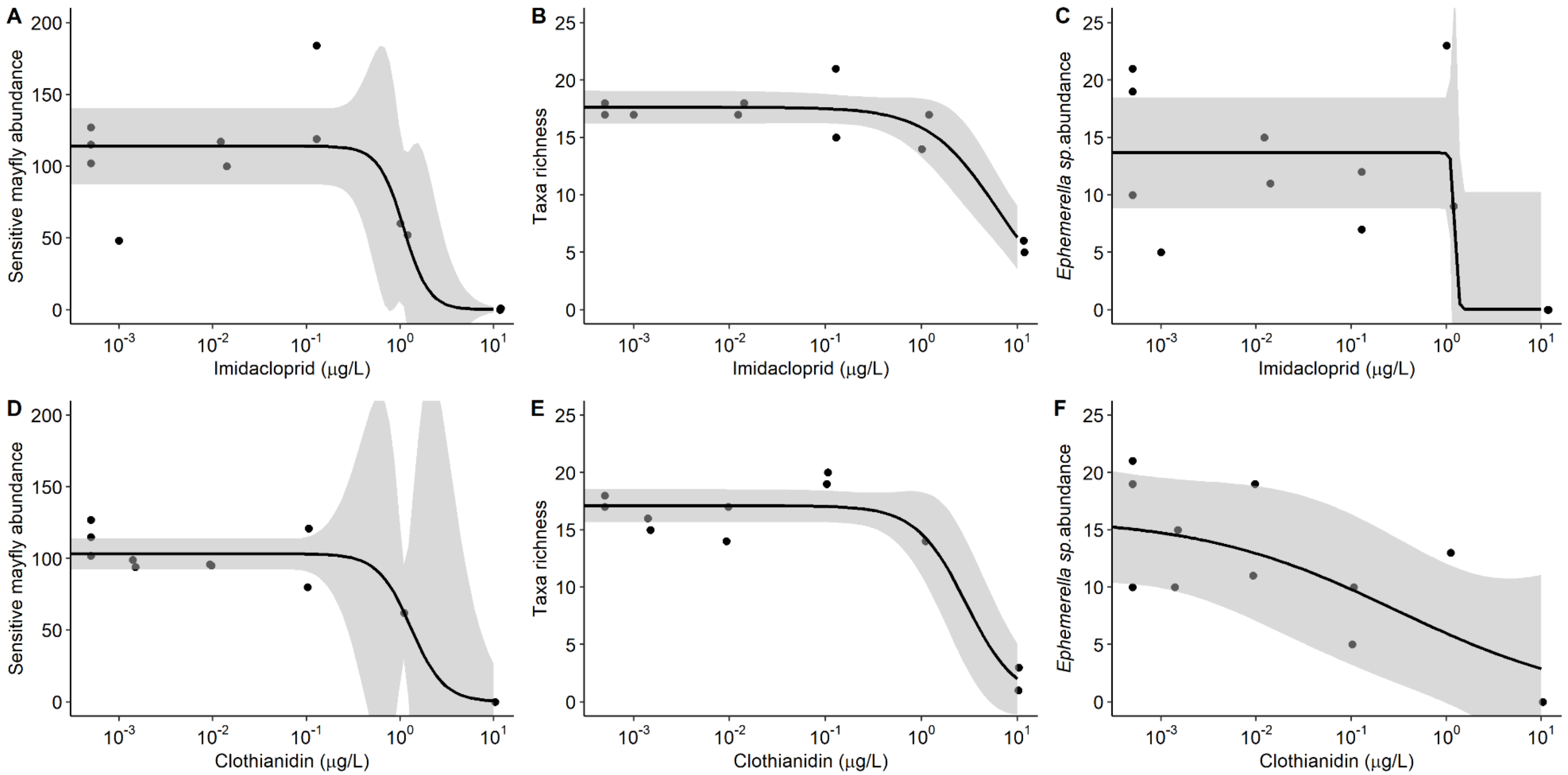

Fig. S3. Larvae abundance as a function of measured imidacloprid (A–C) or clothianidin (D–F) concentration fitted with a 3-parameter logistic function. Each data point represents an individual observation from an experimental stream. Response variables are sensitive mayfly abundance (A, D), taxa richness (B, E), and *Ephemerella spp.* abundance (C, F). Black line, regression line; gray ribbon, 95% confidence interval.

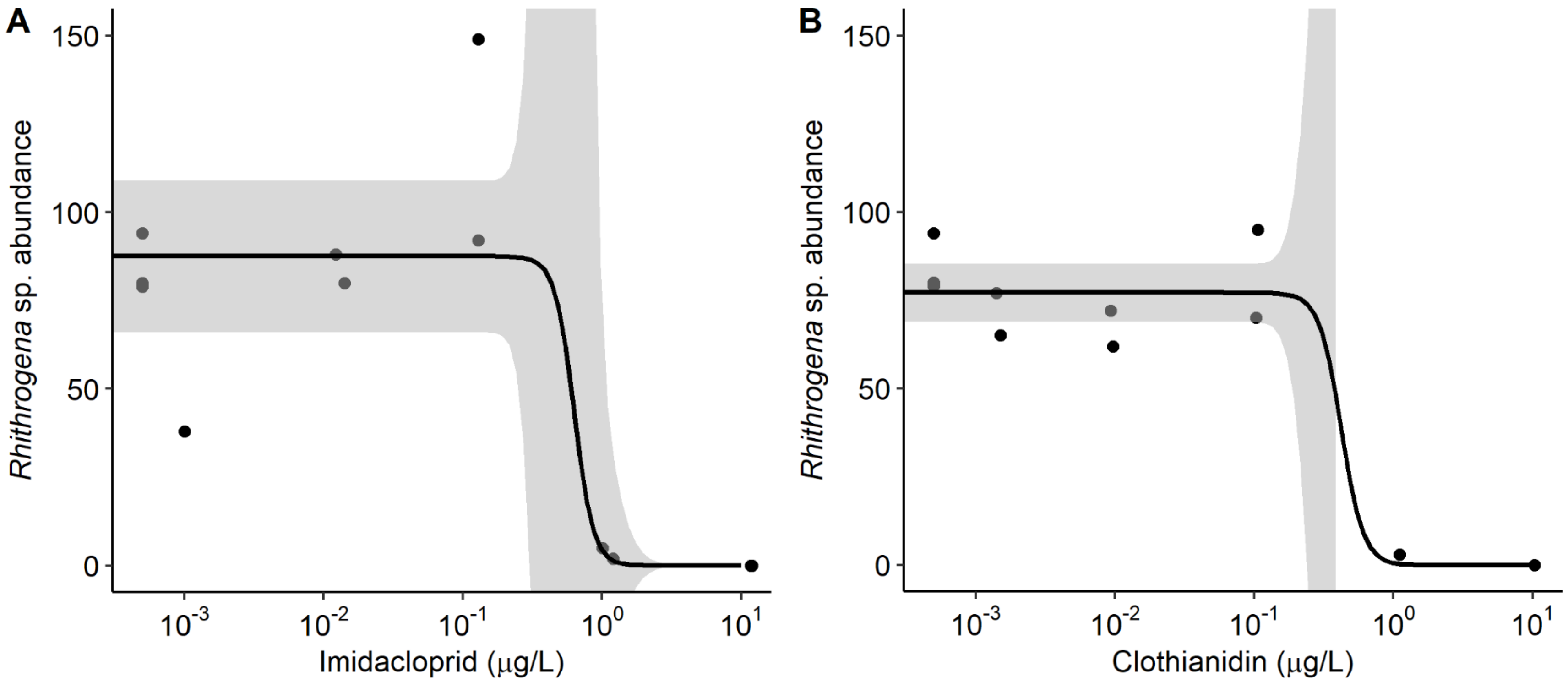

Fig. S4. ***Rhithrogena*** **species (larvae) abundances as a function of measured imidacloprid (A) and clothianidin (B) concentration fitted with a 3-parameter logistic function. Each data point represents an observation from an individual mesocosm. X-axes are log scale. Black line, regression line; gray ribbon, 95% confidence interval.**

203

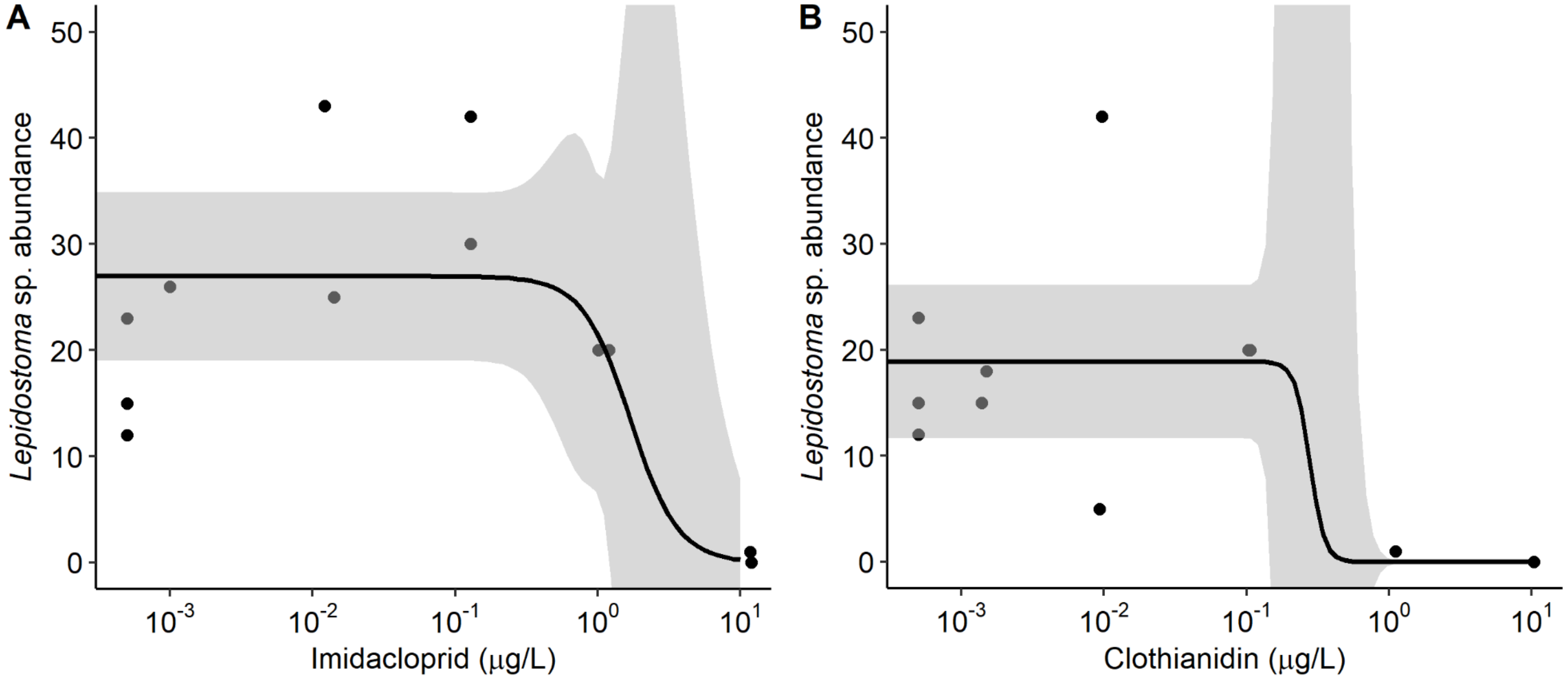

Fig. S5. *Lepidostoma* species (larvae) abundances as a function of measured imidacloprid (A) and clothianidin (B) concentration fitted with a 3-parameter logistic function. Each data point represents an observation from an individual mesocosm. X-axes are log scale. Black line, regression line; gray ribbon, 95% confidence interval.

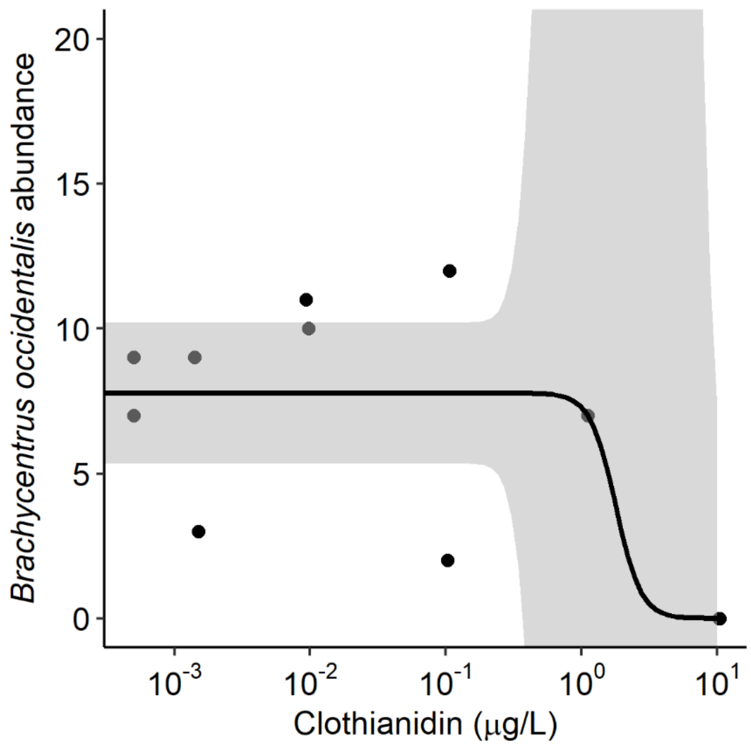

Fig. S6. ***Brachycentrus occidentalis*** **(larvae) abundances as a function of measured clothianidin concentration fitted with a 3-parameter logistic function. Each data point represents an observation from an individual mesocosm values. X-axes are log scale. Black line, regression line; gray ribbon, 95% confidence interval.**

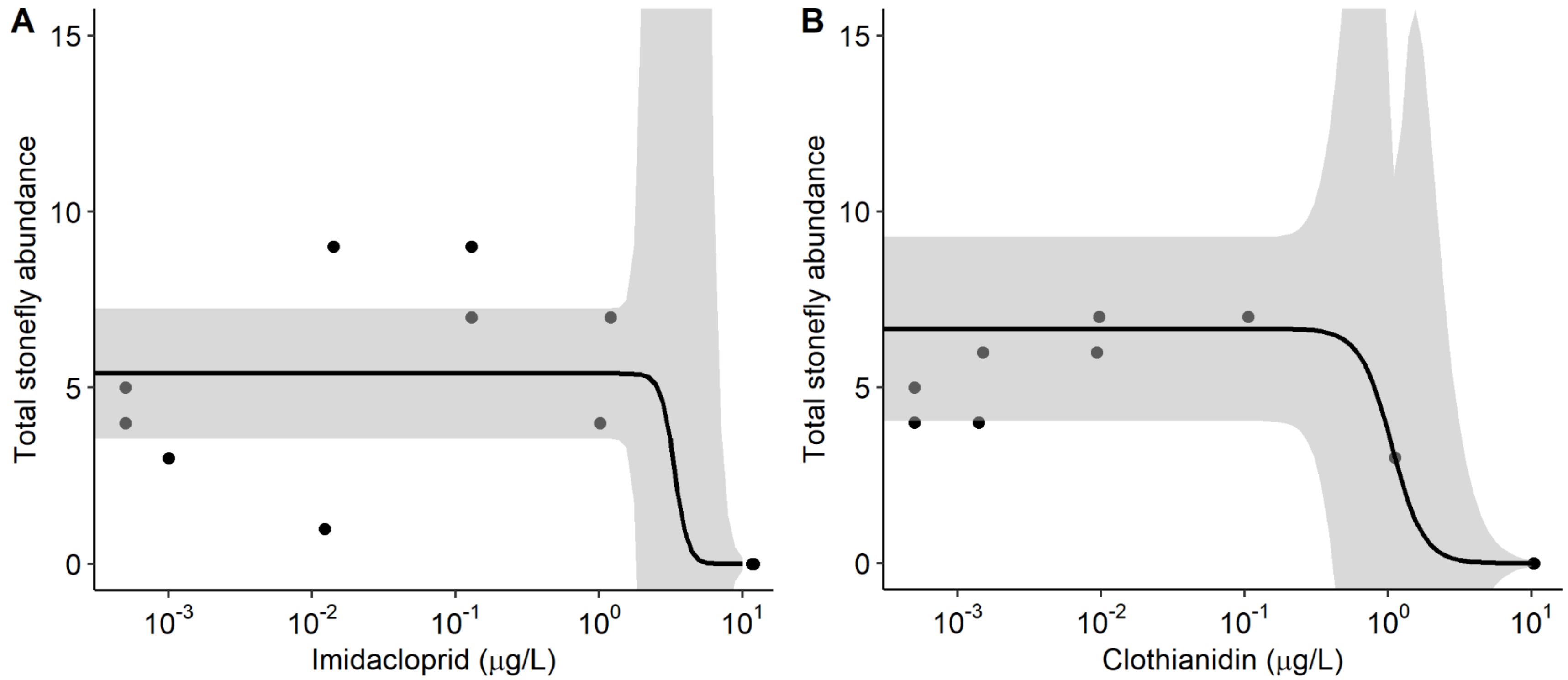

Fig. S7. Total stonefly (larvae) abundances as a function of measured imidacloprid (A) and clothianidin (B) concentration fitted with a 3-parameter logistic function. Each data point represents an observation from an individual mesocosm. X-axes are log scale. Black line, regression line; gray ribbon, 95% confidence interval.

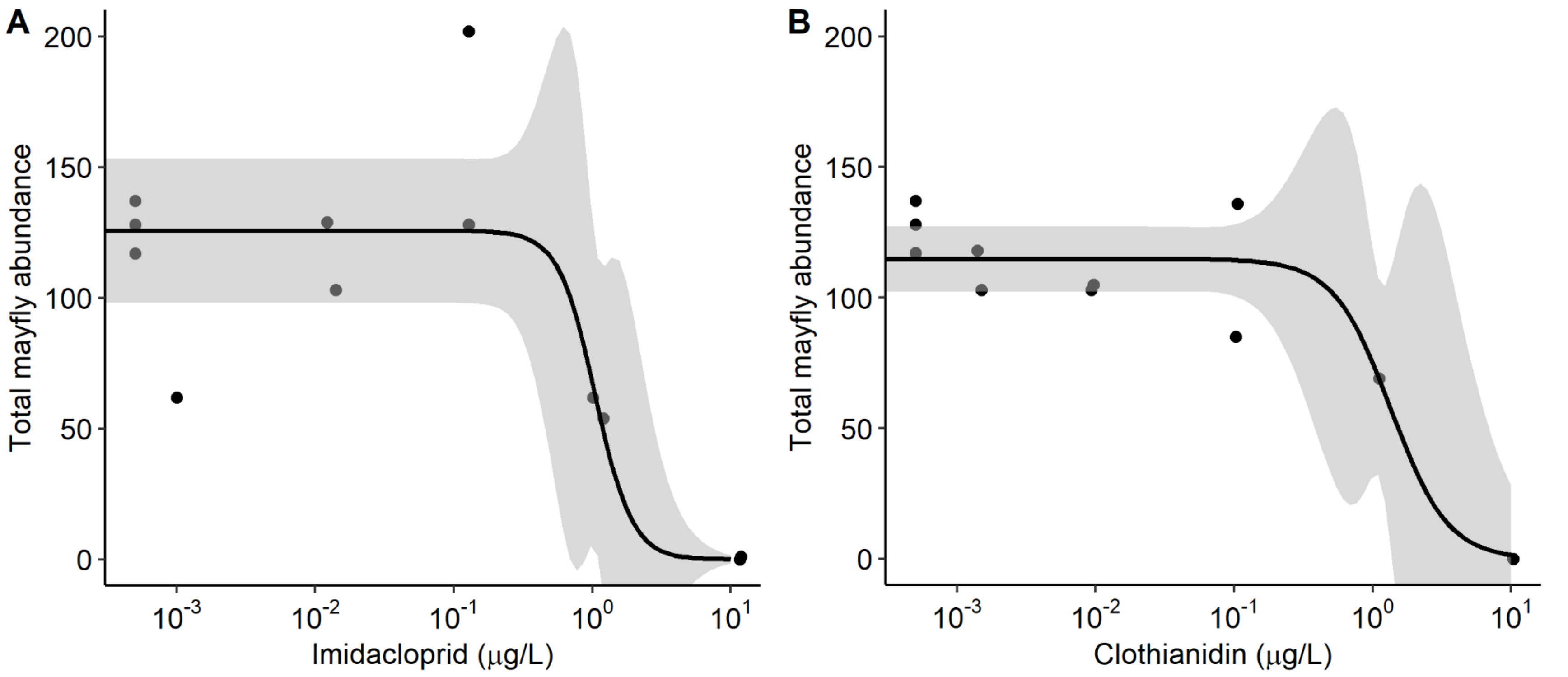

Fig. S8. Total mayfly (larvae) abundances as a function of measured imidacloprid (A) and clothianidin (B) concentration fitted with a 3-parameter logistic function. Each data point represents an observation from an individual stream. X-axes are log scale. Black line, regression line; gray ribbon, 95% confidence interval.

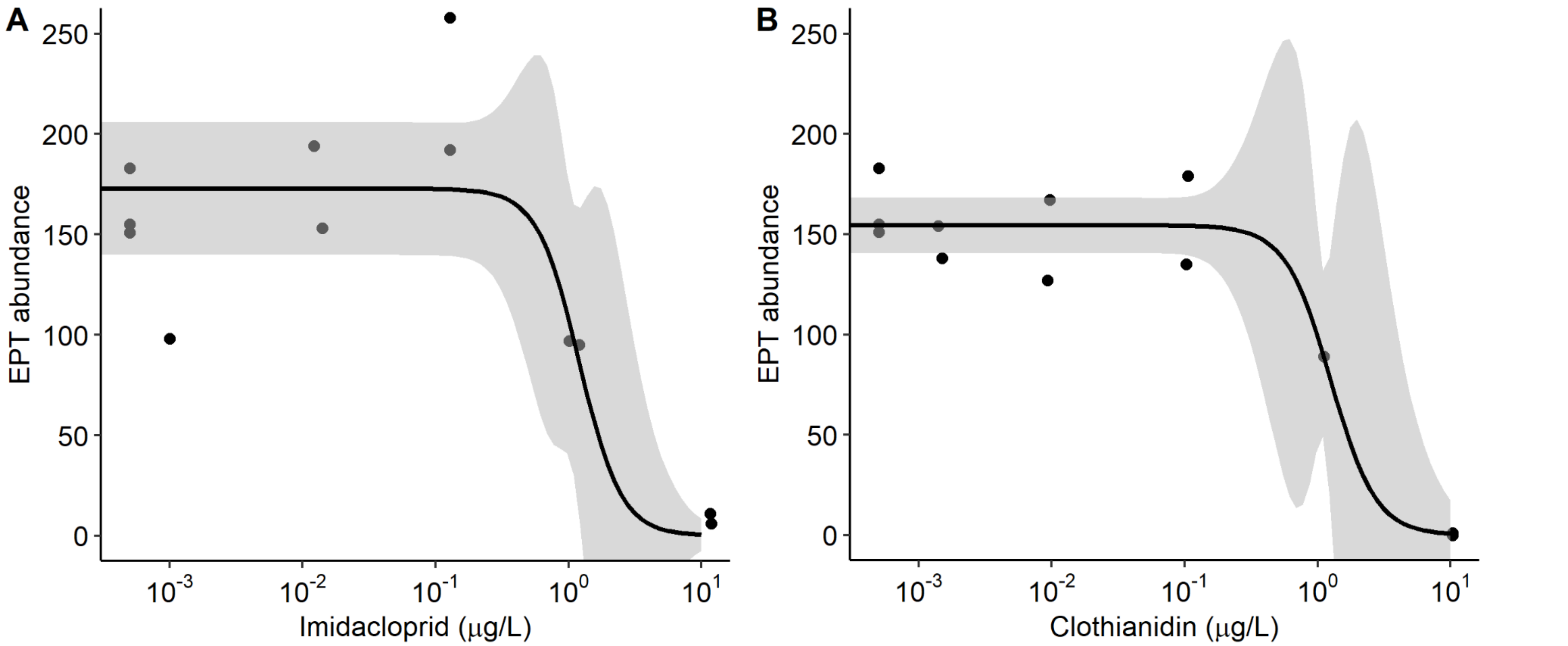

Fig. S9. Ephemeroptera + Plecoptera + Trichoptera (EPT) abundances (larvae) as a function of measured imidacloprid (A) and clothianidin (B) concentration fitted with a 3-parameter logistic function. Each data point represents an observation from an individual stream. X-axes are log scale. Black line, regression line; gray ribbon, 95% confidence interval.

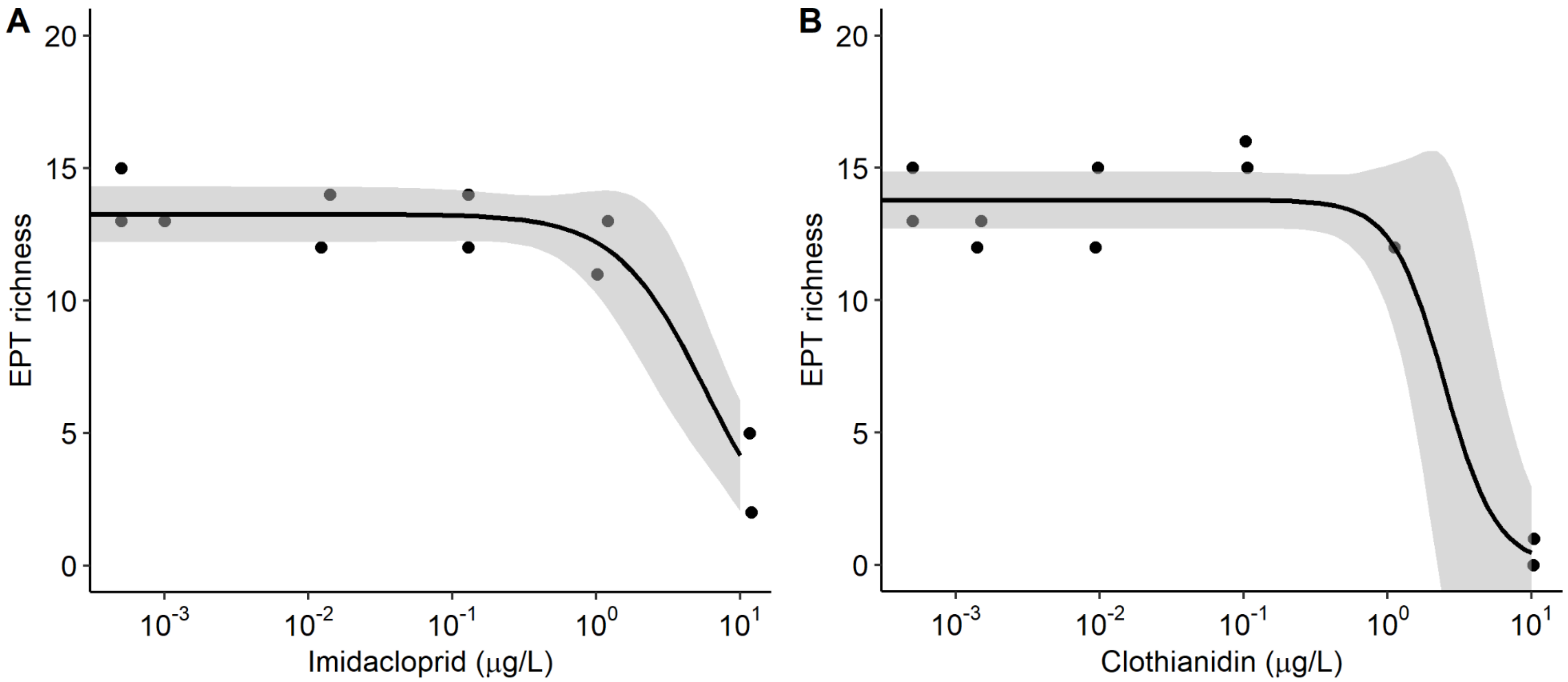

Fig. S10. Ephemeroptera + Plecoptera + Trichoptera (EPT) richness (larvae) as a function of measured imidacloprid (A) and clothianidin (B) concentration fitted with a 3-parameter logistic function. Each data point represents an observation from an individual stream. X-axes are log scale. Black line, regression line; gray ribbon, 95% confidence interval.

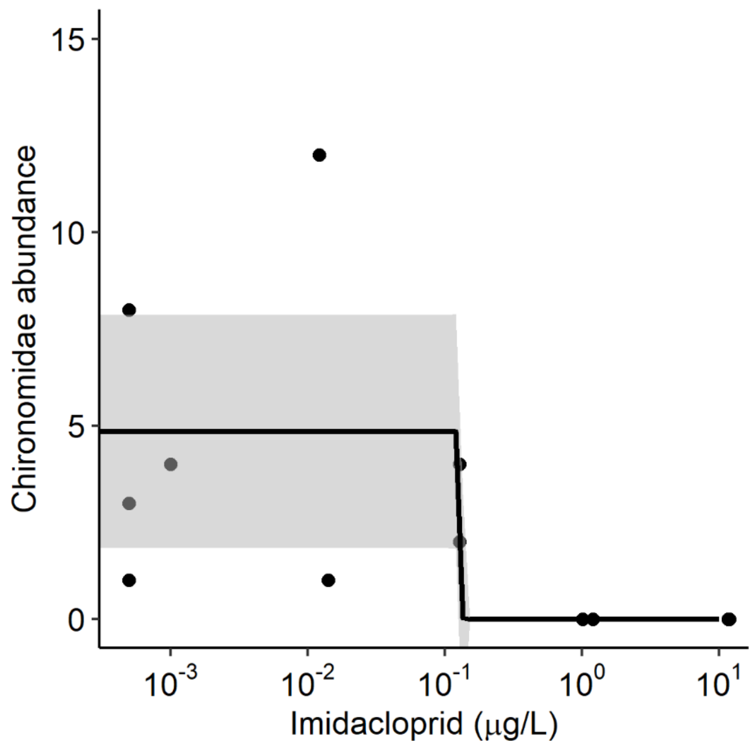

Fig. S11. Chironomidae abundance (larvae) as a function of measured imidacloprid concentration fitted with a 3-parameter logistic function. Each data point represents an observation from an individual stream. X-axes are log scale. Black line, regression line; gray ribbon, 95% confidence interval.

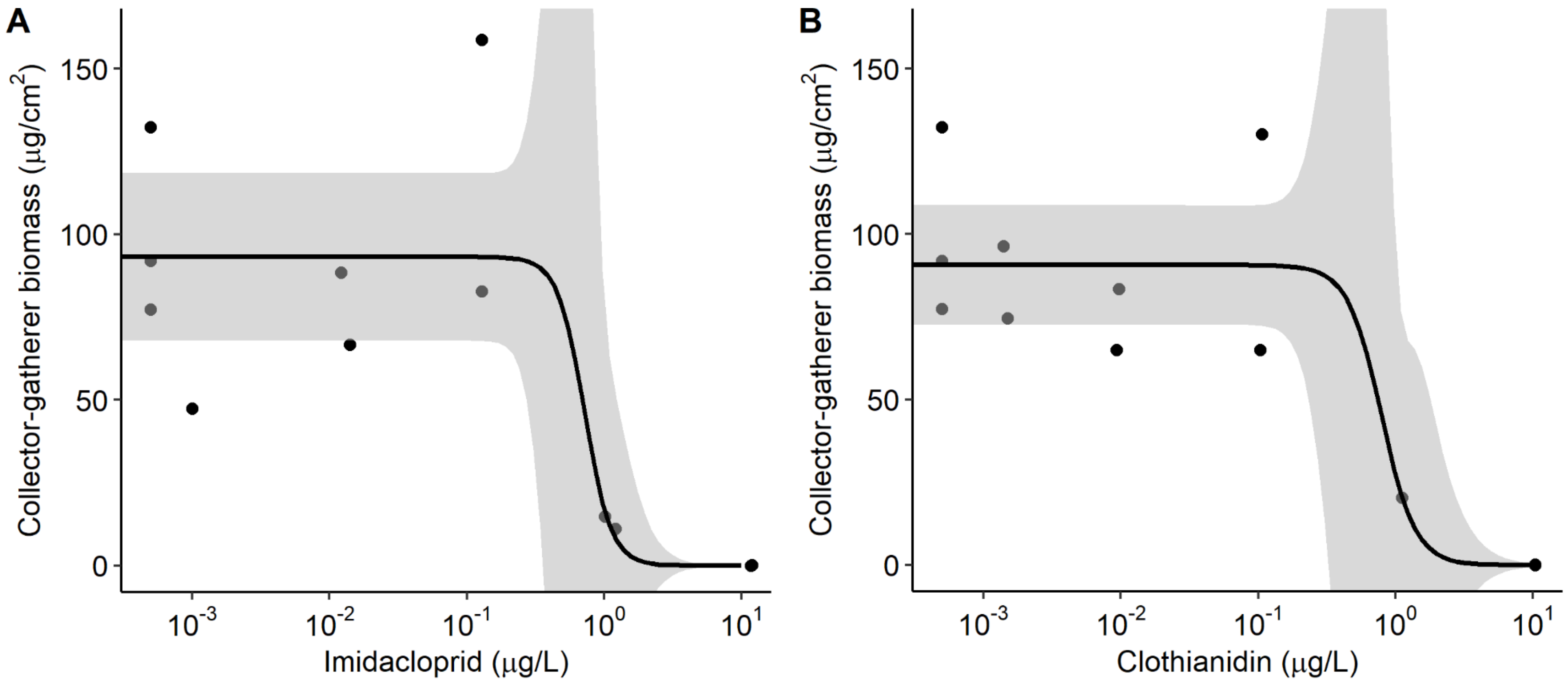

Fig. S12. Collector-gatherer biomass (µg/cm^2) as a function of measured imidacloprid (A) and clothianidin (B) concentration fitted with a 3-parameter logistic function. Each data point represents an observation from an individual mesocosm. X-axes are log scale. Black line, regression line; gray ribbon, 95% confidence interval.

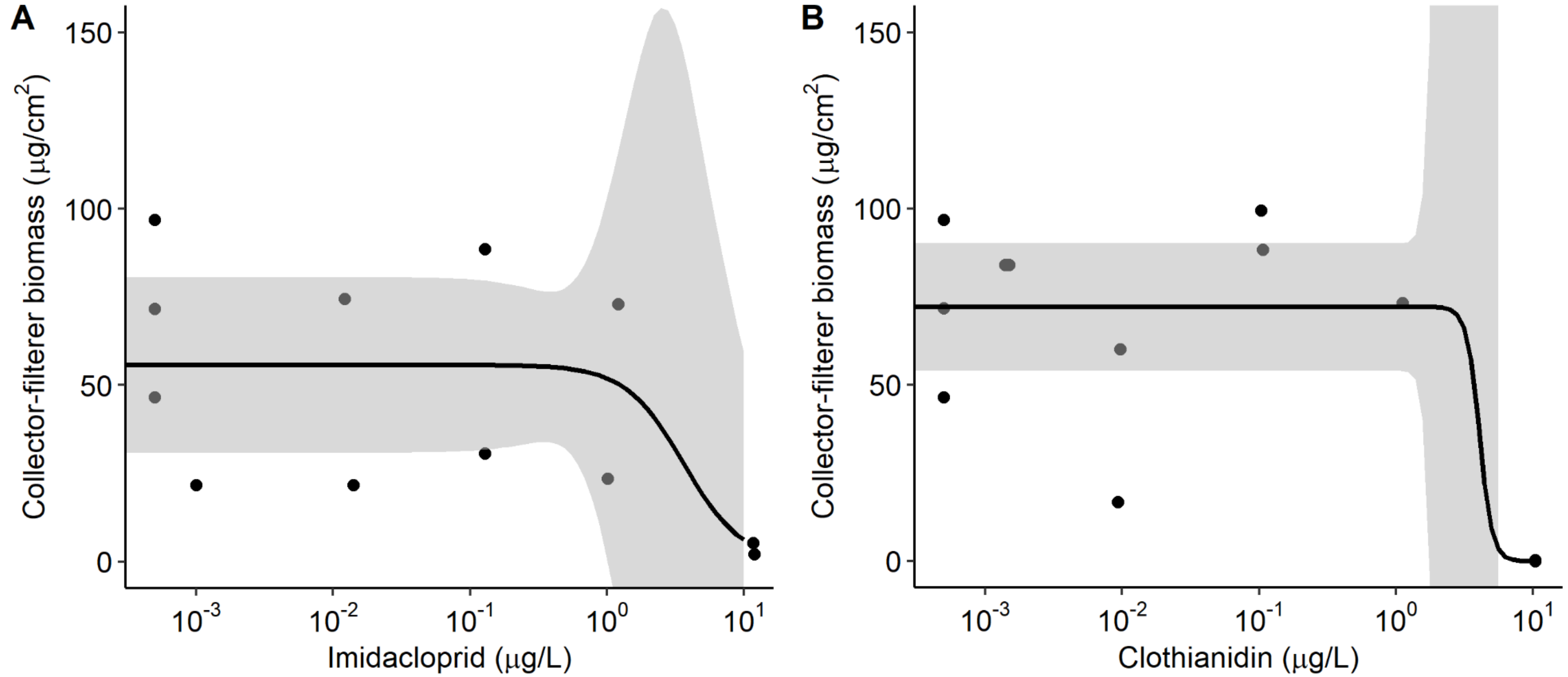

Fig. S13. Collector-filterer biomass (µg/cm^2) (larvae) as a function of measured imidacloprid (A) and clothianidin (B) concentration fitted with a 3-parameter logistic function. Each data point represents an observation from an individual mesocosm. X-axes are log scale. Black line, regression line; gray ribbon, 95% confidence interval.

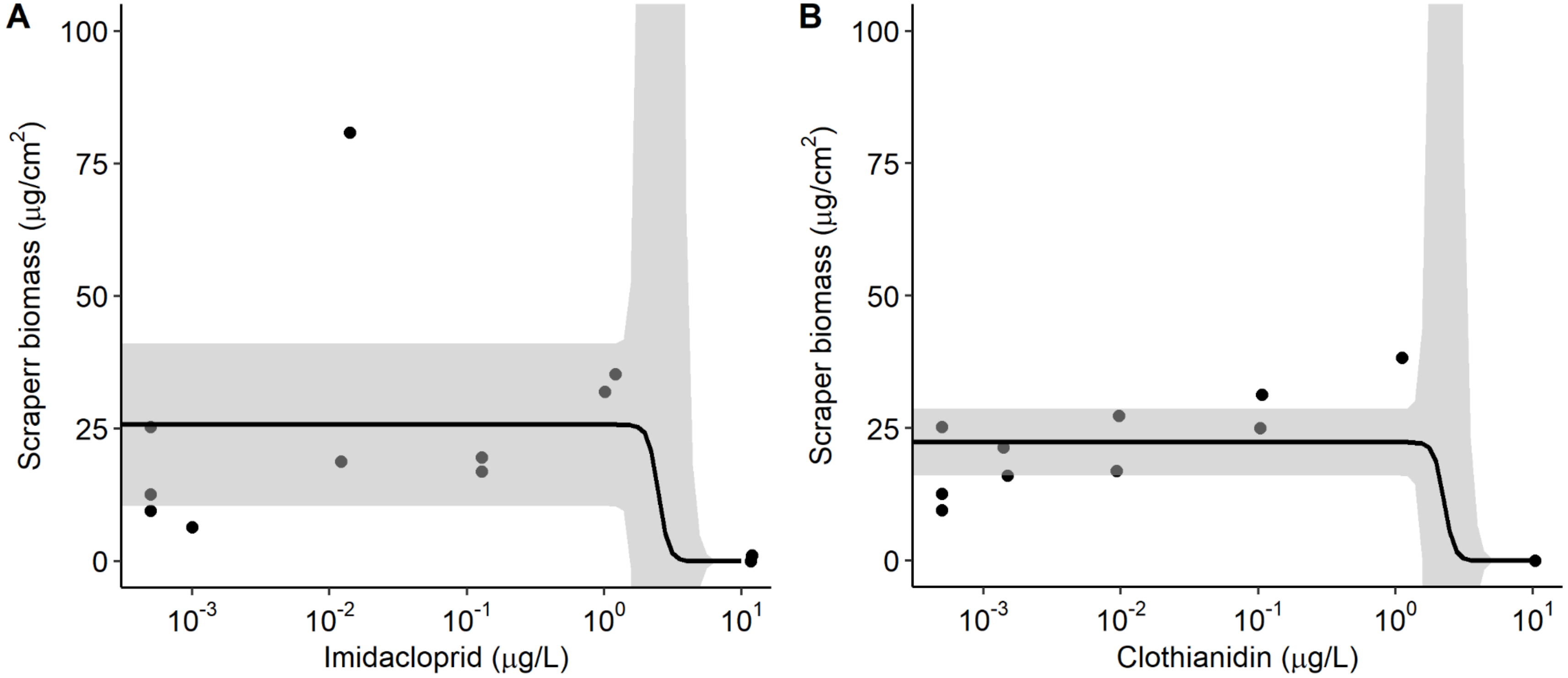

Fig. S14. Scraper biomass (µg/cm²) (larvae) as a function of measured imidacloprid (A) and clothianidin (B) concentration fitted with a 3-parameter logistic function. Each data point represents an observation from an individual mesocosm. X-axes are log scale. Black line, regression line; gray ribbon, 95% confidence interval.

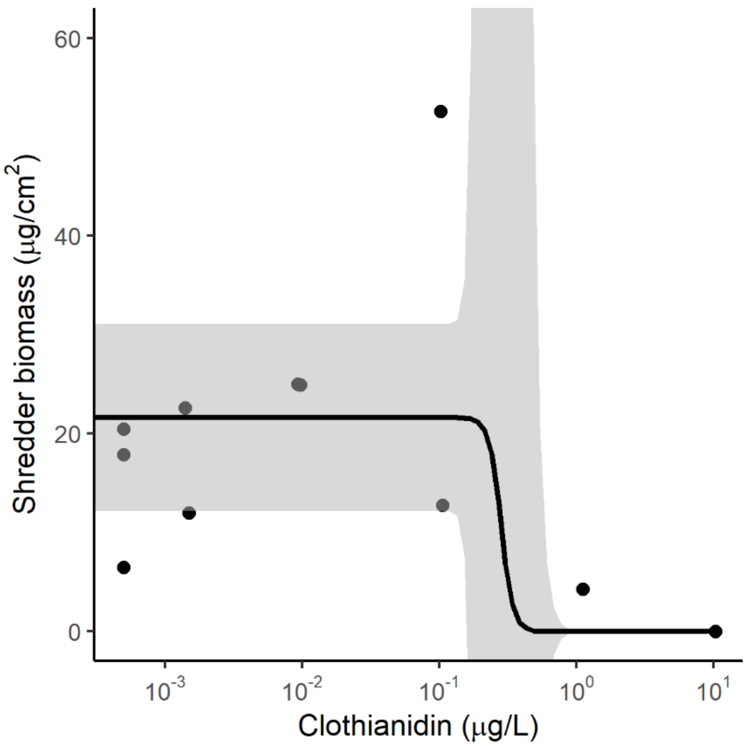

Fig. S15. Shredder biomass (μg/cm^2) (larvae) as a function of measured clothianidin concentration fitted with a 3-parameter logistic function. Each data point represents an observation from an individual mesocosm. X-axes are log scale. Black line, regression line; gray ribbon, 95% confidence interval.

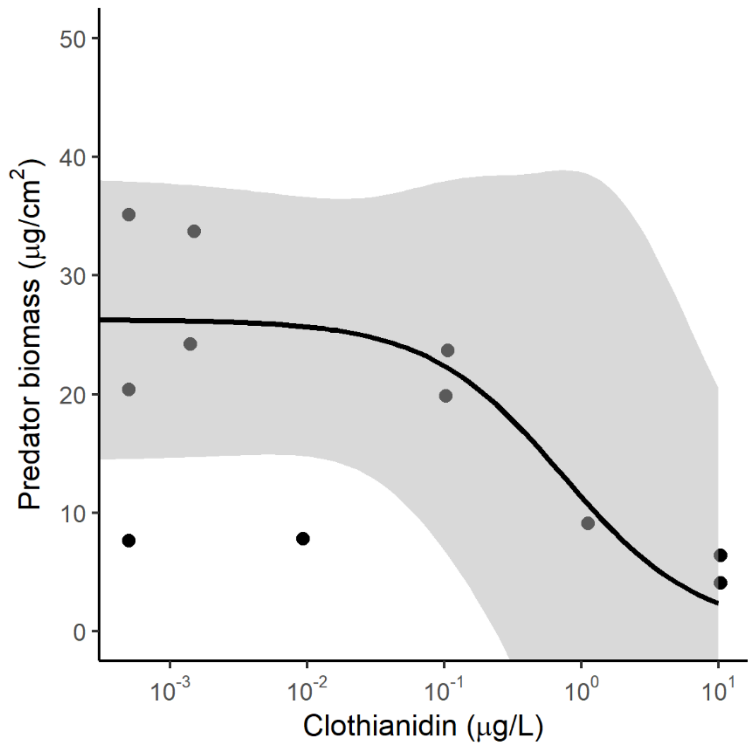

Fig. S16. Predator biomass (µg/cm²) (larvae) as a function of measured clothianidin concentration fitted with a 3-parameter logistic function. Each data point represents an observation from an individual mesocosm. X-axes are log scale. Black line, regression line; gray ribbon, 95% confidence interval.

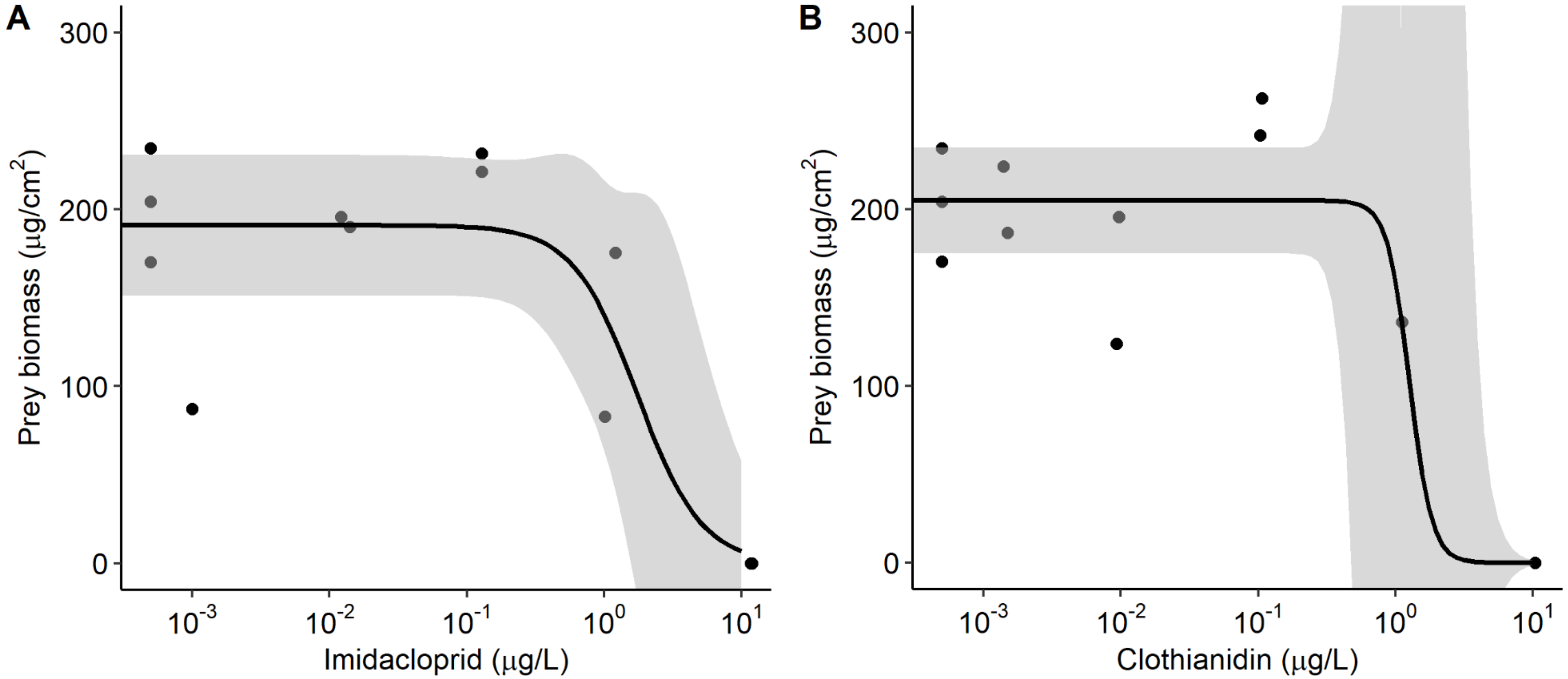

Fig. S17. Prey biomass (mg) (larvae) as a function of measured imidacloprid (A) and clothianidin (B) concentration fitted with a 3-parameter logistic function. Each data point represents an observation from an individual mesocosm. X-axes are log scale. Black line, regression line; gray ribbon, 95% confidence interval.

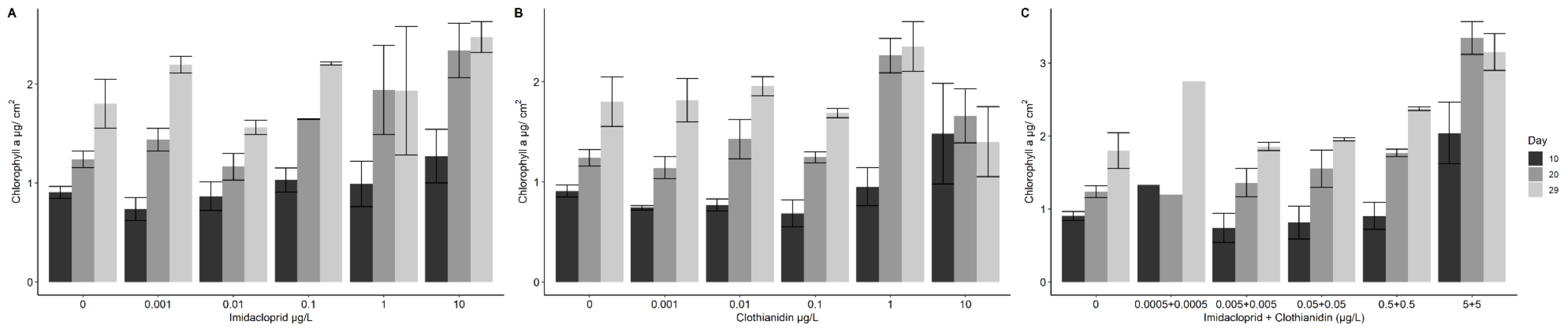

Fig. S18. Chlorophyll *a* concentration (µg/cm^2) as a function of nominal neonicotinoid concentrations for (A) imidacloprid, (B) clothianidin, and (C) 1:1 mixtures of imidacloprid and clothianidin. For each stream, three chlorophyll *a* measurements were taken with a Benthotorch on sampling days 10, 20, and 29. For each bar on the plot, the three replicates were averaged for each stream and chlorophyll *a* concentrations were then averaged across streams of the same nominal concentration.

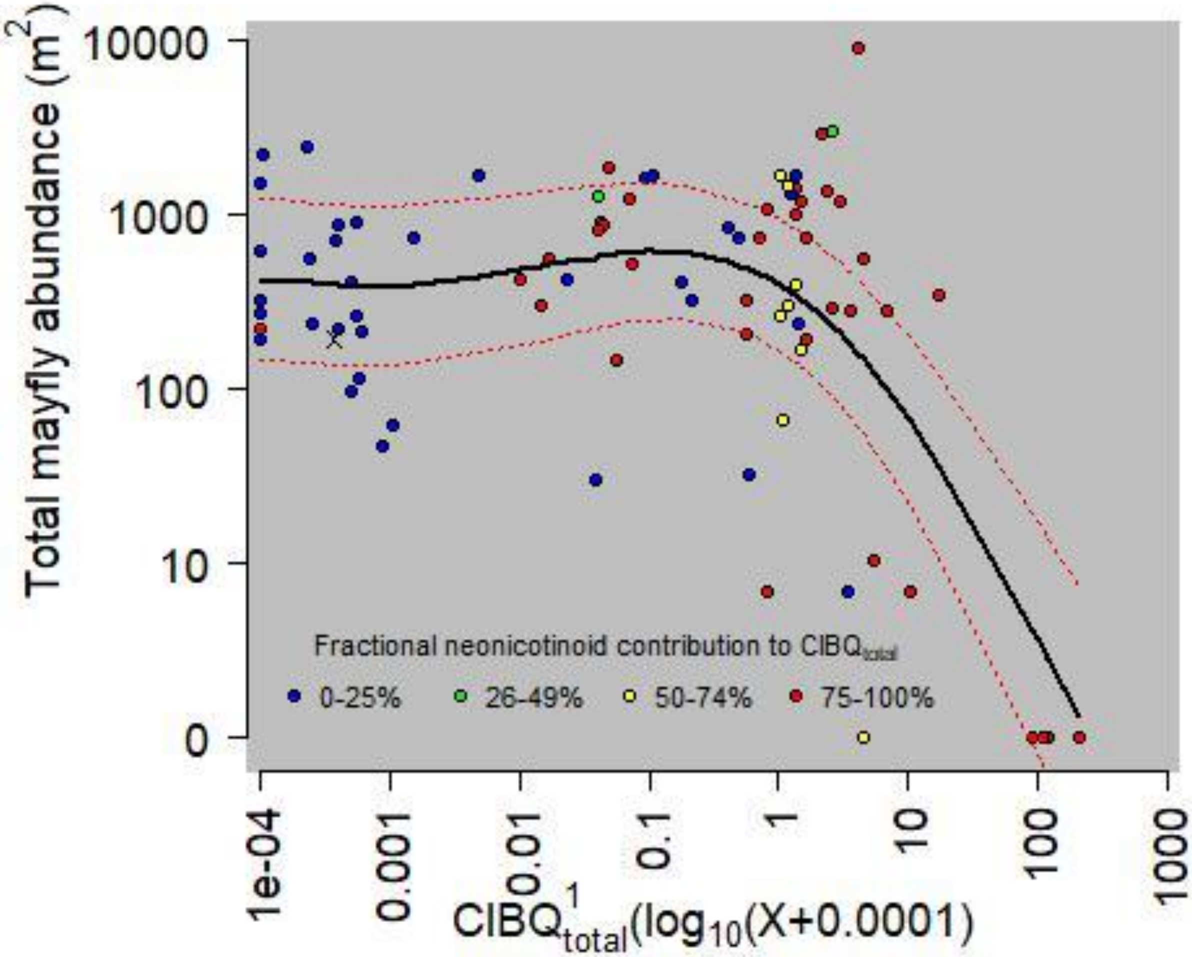

Fig S19. Total mayfly abundance relation to the Chronic invertebrate benchmark quotient ($CIBQ_{total}$). The generalized additive model depicted includes the following covariates: Site elevation- recorded in meters, Average soil bulk density- average across the watershed, Summer average precipitation- June-September 1971-2000 monthly average precipitation across the watershed in millimeters. [1]$CIBQ_{total}$ is calculated by dividing the measured concentration for every pesticide detected in a sample by its chronic invertebrate benchmark (usually the No Observable Adverse Effect Concentration (*9*)), summing them for each sample, and taking the maximum value of the 4 samples observed at each site. CIBQ values are color coded to depict the percentage of CIBQ due to neonicotinoid compounds. Black lines are the generalized additive model (GAM); red dashed lines are 95% confidence intervals of GAM. Black X indicates outlier removed during the development of the covariate only model.

Table S1. Larval aquatic invertebrate effect concentrations (± SE; in µg/L) at the end of 30-day exposures to imidacloprid and clothianidin in a mesocosm experiment. All calculated effects are for larvae except percent emergence. EPT is the sum of Ephemeroptera + Plecoptera + Tricoptera. Sensitive mayfly abundance is the sum of *Ephemerella* spp. + *Rhithrogena* spp. + *Drunella* spp. + *Epeorus* spp. abundances. Prey biomass is the sum of biomass of all invertebrates not considered predators. (EC_{20}, 20% effect concentration EC_{50}, 50% effect concentration; NSC, Nash-Sutcliff Coefficient, similar to coefficient of determination; NA, no effect concentrations determined.

Taxa	Compound	EC_{20}	EC_{50}	NSC
Ephemerella spp.	Imidacloprid	1.17 ± 0.10	1.23 ± 0.12	0.50
	Clothianidin	0.01 ± 0.04	0.30 ± 1.10	0.58
	Mixture[1]	**0.08 ± 0.28**	**0.62 ± 2.53**	0.43
Rhithrogena spp.	Imidacloprid	0.50 ± 2.14	0.63 ± 1.91	0.75
	Clothianidin	0.33 ± 0.40	0.42	0.92
	Mixture[1]	**0.38**	**0.47**	0.91
Lepidostoma spp.	Imidacloprid	0.99 ± 0.62	1.69 ± 1.89	0.56
	Clothianidin	0.24 ± 4.12	0.28 ± 5.70	0.48
	Mixture[1]	**0.58 ± 1.11**	**0.93 ± 0.68**	0.41
Brachycentrus occidentalis	Imidacloprid	NA	NA	NA
	Clothianidin	1.32 ± 29.24	1.78 ± 106.59	0.52
	Mixture[1]	0.04 ± 0.10	0.93 ± 1.61	0.43
Taxa richness	Imidacloprid	1.96 ± 0.97	6.15 ± 1.67	0.90
	Clothianidin	1.28 ± 0.56	2.95 ± 1.04	0.92
	Mixture[1]	0.38 ± 0.28	1.92 ± 0.98	0.80
Total stonefly abundance	Imidacloprid	2.89 ± 61.92	3.34 ± 82.11	0.45
	Clothianidin	0.75 ± 1.38	1.06 ± 0.60	0.42
	Mixture[1]	0.13 ± 0.18	0.44 ± 0.43	0.58
Total mayfly abundance	Imidacloprid	0.69 ± 0.46	1.05 ± 0.27	0.72
	Clothianidin	0.70 ± 0.58	1.35 ± 0.52	0.90
	Mixture[1]	0.94 ± 0.19	1.02 ± 0.09	0.92
Sensitive mayfly abundance	Imidacloprid	0.71 ± 0.48	1.09 ± 0.29	0.70
	Clothianidin	0.74 ± 0.97	1.31 ± 0.69	0.91
	Mixture[1]	0.86 ± 0.40	1.00 ± 0.20	0.89
EPT abundance	Imidacloprid	0.73 ± 0.38	1.21 ± 0.35	0.75
	Clothianidin	0.73 ± 0.57	1.26 ± 0.36	0.93
	Mixture[1]	0.94 ± 0.12	1.04 ± 0.06	0.96
EPT richness	Imidacloprid	2.14 ±1.08	5.72 ± 1.58	0.91
	Clothianidin	1.39 ± 0.52	2.47 ± 1.14	0.94
	Mixture[1]	0.55 ± 0.36	1.73 ± 0.77	0.82
Chironomidae abundance	Imidacloprid	0.13 ± 0.01	0.13 ± 0.005	0.37
	Clothianidin	NA	NA	NA
	Mixture[1]	0.35 ± 4.86	0.43 ± 6.65	0.41
Collector-gatherer biomass	Imidacloprid	0.53 ± 0.77	0.72 ± 0.62	0.69
	Clothianidin	0.53 ± 0.94	0.79 ± 0.70	0.76
	Mixture[1]	0.33 ± 2.34	0.38 ± 1.35	0.65
Collector-filterer biomass	Imidacloprid	0.61 ± 2.68	1.29 ± 1.88	0.36
	Clothianidin	2.05 ± 28.12	2.39 ± 39.16	0.60
	Mixture[1]	0.01 ± 0.03	0.19 ± 0.46	0.59
Scraper biomass	Imidacloprid	2.21 ± 29.33	2.48 ± 38.04	0.20
	Clothianidin	2.00 ± 7.93	2.25 ± 10.38	0.54
	Mixture[1]	1.34	1.51	0.35

Shredder biomass	Imidacloprid	NA	NA	NA
	Clothianidin	0.25 ± 4.14	0.28 ± 5.41	0.39
	Mixture[1]	0.59 ± 1.34	0.90 ± 0.78	0.45
Predator biomass	Imidacloprid	NA	NA	NA
	Clothianidin	0.15 ± 0.35	0.73 ± 1.50	0.32
	Mixture[1]	NA	NA	
Prey biomass	Imidacloprid	0.82 ± 0.48	1.73 ± 1.01	0.73
	Clothianidin	0.98 ± 2.32	1.26 ± 2.90	0.83
	Mixture[1]	0.59 ± 1.21	0.82 ± 0.83	0.80
Percent emergence	Imidacloprid	0.053 ± 0.154	2.63 ± 2.66	0.59
	Clothianidin	NA	0.13 ± 0.30	0.52
	Mixture[1]	0.25 ± 0.46	**2.60 ± 3.03**	0.48

[1]Effect concentrations reported for mixtures indicate the sum concentration of imidacloprid and clothianidin. Gray filled cells indicate where mixture effect concentration estimates are lower than unary effect concentration estimates. Bold numbers indicate where mixture effect concentration estimates are intermediate to unary effect estimates.

Table S2. Optimum dynamic multiple reaction monitoring parameters for the determination of pesticide compounds by direct aqueous-injection liquid chromatography-tandem mass spectrometry. [Table is sorted by retention time; for each pesticide compound, the first multiple reaction monitoring (MRM) precursor and product ion pair listed is the quantifier (precursor) ion and the second MRM is the qualifier (product) ion; da, Dalton; min, minutes; CASRN, Chemical Abstracts (CAS) Registry Number®; m/z, mass to charge; V, volts; CE, collision energy; Acc, accelerator]

Retention time (min)	Compound name	CASRN	Nominal mass (da)	Molecular ion	Precursor ion (m/z)	Product ion (m/z)	CE (V)	Cell Acc (V)
7.02	Imidacloprid	138261-41-3	255.1	[M+H]+	256.1	175.1	16	4
						209.0	8	4
7.02	Imidacloprid-d_4	1015855-75-0	259.1	[M+H]+	260.1	179.7	20	7
						213.4	12	7
7.3	Clothianidin-d_3	1262776-24-8	252	[M+H]+	253	132.3	20	2
						172.4	12	2
7.36	Clothianidin	210880-92-5	249	[M+H]+	250	132.4	12	4
						169.6	12	4

Table S3. Summary of recovery of internal standards (imidacloprid-d_4, clothianidin-d_3,) determined in laboratory quality control (QC) samples and mesocosm stream samples (Sample) by direct aqueous-injection liquid chromatography-tandem mass spectrometry. [USGS, U.S. Geological Survey; QC, quality control; ng/L, nanograms per liter; RSD, relative standard deviation]

Compound name	Sample Type	Expected concentration (ng/L)	Recovery, in percent					
			Number of samples	Mean	RSD	Median	Minimum	Maximum
Imidacloprid-d_4	QC	400	69	101	7.7	99.81	85.8	128
	Sample	400	117	104.1	6.9	103.5	89.3	131
	All		186	103	7.3	102.5	85.8	131
Clothianidin-d_3	QC	750	69	101.5	6.8	100.2	83.8	122
	Sample	750	117	105.2	7.8	104.8	84.7	127
	All		186	103.8	7.6	103.1	83.8	127

Table S4. Summary of detection frequency and concentrations of pesticide compounds determined in laboratory quality control blank samples by direct aqueous-injection liquid chromatography-tandem mass spectrometry.
[Samples were analyzed in two batches containing five CCB and one PBLNK sample each; USGS, U.S. Geological Survey; QC, quality control; ng/L, nanograms per liter, CCB, continuing calibration instrument blank sample; PBLNK, laboratory reagent blank sample]

Compound Name	QC Sample name	Number of samples	Sum of detections	Detection frequency (percent)	Median concentration (ng/L)	Maximum concentration (ng/L)
Imidacloprid	CCB	9	0	0	0	0
	PBLNK	2	0	0	0	0
CCB6	1	1	100	38	38	
Clothianidin	CCB	9	0	0	0	0
	PBLNK	2	0	0	0	0
	CCB6	1	1	100	17.5	17.5

Note: CCB6 blank sample analyzed at the end of one batch had carryover from diluted nominal spike samples (100 mg/L at 50X dilution) analyzed in the sequence immediately before that sample.

Table S5 Recovery of pesticide compounds in laboratory quality control spike samples determined in two analytical batches of mesocosm samples by direct aqueous-injection liquid chromatography-tandem mass spectrometry. [USGS, U.S. Geological Survey; QC, quality control; ng/L, nanograms per liter; CCV, continuing calibration verification sample; IDL, instrument detection level sample; MDL, method detection level; PSPK, laboratory reagent spike sample; RSD, relative standard deviation]

Sample Type	Compound name	USGS Parameter method code	QC Sample name	Expected concentration (ng/L)	MDL (ng/L)	Number of samples	Recovery, in percent				
							Mean	RSD	Median	Minimum	Maximum
QC	Imidacloprid	68426LCM75	CCV	250	7	9	107.6	7	108.6	96.5	122
			IDL	5	7	1	38.29	.	38.29	38.3	38.3
				10	7	1	93.2	.	93.2	93.2	93.2
				25	7	2	106.6	16	106.6	94.7	119
				50	7	2	91.78	19	91.78	79.5	104
			PSPK	250	7	3	102.7	3	101.8	100	106
			All		7	18	100.3	18	103.4	38.3	122
QC	Clothianidin	68221LCM75	CCV	250	3	9	110.1	8	107.1	103	130
			IDL	5	3	1	80.05	.	80.05	80.1	80.1
				10	3	1	103.9	.	103.9	104	104
				25	3	2	111.7	3	111.7	110	114
				50	3	2	90.81	7	90.81	86.3	95.3
			PSPK	250	3	3	95.78	2	95.51	94.3	97.5
			All		3	18	103.7	11	103.2	80.1	130

Note: CCV6 and the 5- and 10-ng/L IDL samples analyzed at the end of one batch had potential carryover from diluted nominal spike samples (100 mg/L at 50X dilution) analyzed in the sequence immediately before these samples and were excluded from this summary.

Table S6. Model-fit statistics used for selecting the best-fit data distribution used to estimate Hazard Concentrations at the 5th percentile (HC_5) of each species sensitivity distribution shown in Fig. 4. Each distribution fit to the data was used to estimate an HC_5 that was then weighted by AIC_c weight to derive mean HC_5 values for imidacloprid and clothianidin.

Compound	Distribution	Anderson-Darling	Kolmogorov-Smirnov	Cramer-von Mises	Akaike's Information Criterion (AIC)	AIC corrected (AIC_c)	Bayesian Information Criterion	$\Delta_i AIC$	$w_i(AIC_C)$
**Imidacloprid**	lnorm	0.862	0.255	0.158	48.9	49.8	50.5	0.386	0.323
	lgumbel	1.14	0.278	0.215	52.1	53.0	53.6	3.550	0.066
	gamma	0.679	0.175	0.103	49.7	50.6	51.2	1.160	0.219
	weibull	0.598	0.196	0.0974	48.5	49.5	50.1	0.00	0.392
**Clothianidin**	lnorm	0.356	0.162	0.0525	14.4	16.4	14.8	1.650	0.177
	lgumbel	0.511	0.212	0.0844	16.6	18.6	17.0	3.800	0.060
	gamma	0.182	0.128	0.0239	12.8	14.8	13.2	0.000	0.404
	weibull	0.219	0.14	0.0283	13.0	15.0	13.4	0.239	0.359

lnorm-log-normal distribution; lgumbel-log gumbel distribution; gamma-gamma distribution; weibull- weibull distribution. AIC- Akaike Information Criteria. AIC corrected (AIC_c) the model AIC corrected for model complexity and observations expressed as (AIC + (2*k*(k+1))/n-k-1)) where k is the number of model parameters and n is the number of observations. $\Delta_i AIC$ is the difference in the AIC value of models expressed as ($\Delta_i AIC$ - (AIC_i – min(AIC)). $w_i(AIC)$ – exp(-0.5* $\Delta_i AIC)_i$/sum($\Delta_i AIC$)). $w_i(AIC_C)$ weight is the likelihood of the model expressed as (exp(-0.5* $\Delta_i AIC_C$)).

Table S7: Information on the California Stream Quality Assessment study sites, data from Qi and Nakagaki (*10*).

Map key[1]	NWIS site number	USGS station name	Latitude	Longitude
1	383719122462501	MAACAMA C A CHALK HILL RD BR NR HEALDSBURG CA	38.621958	-122.773689
2	11465350	DRY C NR MOUTH NR HEALDSBURG CA	38.58741	-122.862216
3	383321122302101	NAPA R A BALE LN NR DEER PARK CA	38.555946	-122.505915
4	383305122311901	RITCHEY C NR DEER PARK CA	38.551424	-122.521015
5	383109122363301	MARK WEST C A TARWATER RD NR CALISTOGA CA	38.519047	-122.609222
6	383039122502401	WINDSOR C A MARK WEST STATION RD NR WINDSOR CA	38.510957	-122.839956
7	11466800	MARK WEST C NR MIRABEL HEIGHTS CA	38.494001	-122.853097
8	11456500	CONN C NR OAKVILLE CA	38.447278	-122.380556
9	11466320	SANTA ROSA C A WILLOWSIDE RD NR SANTA ROSA CA	38.44519	-122.807213
10	382634122315201	SONOMA C A ADOBE CYN RD NR KENWOOD CA	38.4427	-122.531
11	11466170	MATANZAS C A SANTA ROSA CA	38.438801	-122.702487
12	382619122531401	GREEN VALLEY C A GRATON CA	38.438729	-122.887208
13	11466200	SANTA ROSA C A SANTA ROSA CA	38.43679	-122.724695
14	11465690	COLGAN C NR SANTA ROSA CA	38.402135	-122.733043
15	382346122521201	UNNAMED TRIB A MONTGOMERY RD NR SEBASTOPOL CA	38.396125	-122.870039
16	382245122001601	ULATIS C A FARRELL RD NR VACAVILLE CA	38.379169	-122.004319
17	11465660	COPELAND C A ROHNERT PARK CA	38.343248	-122.701932
18	382035121575501	ALAMO C A TULARE RD BR NR VACAVILLE CA	38.343083	-121.96535
19	382017122161101	MILLIKEN C BL HEDGESIDE AVE NR NAPA CA	38.338155	-122.269874
20	11458500	SONOMA C A AGUA CALIENTE CA	38.323247	-122.494426
21	11458300	NAPA C A NAPA	38.301859	-122.303863
22	381740122395901	LICHAU C A PENNGROVE CA	38.294481	-122.666361
23	381556122280201	SONOMA C A WATMAUGH RD BR NR SONOMA CA	38.265797	-122.467397
24	381519122385601	PETALUMA R NR PETALUMA CA	38.255189	-122.648971
25	381441122064301	SUISUN C A ROCKVILLE CA	38.244797	-122.111997

26	11459500	NOVATO C A NOVATO CA	38.107698	-122.579981
27	380410122315501	ARROYO SAN JOSE A DIGITAL DR NR NOVATO CA	38.069497	-122.531997
28	380345122345201	ARROYO SAN JOSE A FAIRWAY DR NR NOVATO CA	38.062272	-122.581152
29	11182400	ARROYO DEL HAMBRE A MARTINEZ CA	38.00352	-122.129699
30	375819122035801	GRAYSON C A GOLF CLUB RD NR PLEASANT HILL CA	37.972091	-122.066128
31	375807122124001	PINOLE C BL ALHAMBRA VALLEY RD NR PINOLE CA	37.969611	-122.217774
32	375808122172601	WILKIE C A SANTA RITA RD NR RICHMOND CA	37.968842	-122.290553
33	11460000	CORTE MADERA C A ROSS CA	37.962979	-122.556922
34	375746122195501	SAN PABLO C A EL PORTAL DR A SAN PABLO CA	37.9628	-122.331997
35	375701121564401	MT DIABLO C A CLAYTON CA	37.950364	-121.945647
36	375413122033301	WALNUT C A CIVIC DR A WALNUT CREEK CA	37.903669	-122.059217
37	375312122113501	SAN PABLO C A ORINDA CA	37.886553	-122.193119
38	375257122050001	LAS TRAMPAS C A LAFAYETTE CA	37.882397	-122.099997
39	375220122104201	SAN PABLO C A MORAGA WAY A ORINDA CA	37.864508	-122.172306
40	374933122001301	SAN RAMON C A LA GONDA WAY A DANVILLE CA	37.825711	-122.003539
41	374708122132801	SAUSAL C A OAKLAND CA	37.785681	-122.224339
42	11182500	SAN RAMON C A SAN RAMON CA	37.772983	-121.994682
43	374336122095801	SAN LEANDRO C A ALVARADO ST A SAN LEANDRO CA	37.726608	-122.166178
44	11180900	CROW C NR HAYWARD CA	37.704829	-122.044033
45	11181000	SAN LORENZO C A HAYWARD CA	37.685943	-122.064223
46	11181008	CASTRO VALLEY C A HAYWARD CA	37.679931	-122.080519
47	11176900	ARROYO DE LA LAGUNA A VERONA CA	37.62672	-121.882795
48	11179100	ALAMEDA C NR FREMONT CA	37.566602	-122.001628
49	372716122080801	SAN FRANCISQUITO C DS OF NEWELL RD BR A PALO ALTO	37.454383	-122.136631
50	11164500	SAN FRANCISQUITO C A STANFORD UNIVERSITY CA	37.423273	-122.18941
51	372500122081201	MATADERO C A JOSINA AVE A PALO ALTO CA	37.4168	-122.136592
52	372303121542901	COYOTE C BL CHARCOT AVE NR SAN JOSE CA	37.384453	-121.907849

53	11169025	GUADALUPE R ABV HWY 101 A SAN JOSE CA	37.37383	-121.933013
54	371814121525601	GUADALUPE R A WILLOW GLEN WAY NR SAN JOSE CA	37.303947	-121.88225
55	371738121555901	LOS GATOS C A E HAMILTON AVE NR CAMPBELL CA	37.2939	-121.933
56	371620122005801	SARATOGA C A BRAEMAR DR A SARATOGA CA	37.272252	-122.016288
57	371554121474101	COYOTE C A COYOTE RD NR SAN JOSE CA	37.264939	-121.794658
58	11153470	LLAGAS C AB CHESBRO RES NR MORGAN HILL CA	37.148333	-121.768333
59	370512121361901	LLAGAS C A SAN MARTIN AVE A SAN MARTIN CA	37.086742	-121.605206
60	11169800	COYOTE C NR GILROY CA	37.077723	-121.494383
61	365955121350601	UVAS C A MILLER AVE A GILROY CA	36.998533	-121.584958
62	11153650	LLAGAS C NR GILROY	36.987297	-121.527908
63	365736121250801	PACHECO C A SAN FELIPE RD NR DUNNEVILLE CA	36.959818	-121.418968
64	365718121444301	UNNAMED TRIB A PAULSEN RD NR FREEDOM CA	36.955181	-121.745631
65	365634121264001	TEQUISQUITA SLOUGH A SHORE RD NR DUNNEVILLE CA	36.9427	-121.4445
66	11159200	CORRALITOS C A FREEDOM CA	36.939397	-121.770506
67	11159500	PAJARO R A WATSONVILLE CA	36.905297	-121.7514
68	11159000	PAJARO R A CHITTENDEN CA	36.900231	-121.597721
69	11152600	GABILAN C NR SALINAS CA	36.755792	-121.610501
70	11152650	RECLAMATION DITCH NR SALINAS CA	36.704959	-121.704948
71	364155121363901	NATIVIDAD C NR SALINAS CA	36.6987	-121.610722
72	364138121373701	GABILAN C AB E LAUREL DR NR SALINAS CA	36.693963	-121.627252
73	364003121373501	ALISAL C A FAIRVIEW AVE NR SALINAS CA	36.66725	-121.626999
74	363608121255201	CHUALAR C A CHUALAR CANYON RD NR CHUALAR CA	36.6022	-121.430997
75	11152000	ARROYO SECO NR SOLEDAD CA	36.280858	-121.321877
76	11150500	SALINAS R NR BRADLEY CA	35.930219	-120.868574
77	11148900	NACIMIENTO R BL SAPAQUE C NR BRYSON CA	35.788579	-121.093805
78	352934120395501	ATASCADERO C A W MALL BR A ATASCADERO CA	35.492883	-120.665164
79	352127120484501	CHORRO C A CHORRO C RD NR MORRO BAY CA	35.357508	-120.812464

80	351725120395901	STENNER C A MURRAY AVE A SAN LUIS OBISPO CA	35.2904	-120.666319
81	351436120405201	SAN LUIS OBISPO C A LOS OSOS VLY RD NR SAN LUIS OB	35.243	-120.681444
82	11141280	LOPEZ C NR ARROYO GRANDE CA	35.235549	-120.472735
83	11141050	ORCUTT C NR ORCUTT CA	34.88359	-120.494888
84	11135250	SANTA YNEZ R A 13TH ST BRIDGE A VAFB NR LOMPOC CA	34.677687	-120.553867
85	11128500	SANTA YNEZ R A SOLVANG CA	34.584987	-120.144593

[1]Map key corresponds with map locations in Fig. S1. Data for these sites can be accessed at https://doi.org/10.5066/F7P55KJN.

Table S8: Summary of model information used to select the best candidate model for predicting total mayfly abundance in Coastal California streams.

Model	df	AIC_i	$\Delta_i AIC$	$w_i(AIC)$	Adj-R^2
*IMI*CLO maximum concentration interaction model*	32.41	133.12	0.00	0.99	*0.72*
$CIBQ^{\dagger}_{IMI}$ * $CIBQ_{CLO}$ interaction model	30.88	144.9	11.78	0.01	0.66
Maximum IMI concentration	20.01	151.75	18.63	0.00	0.60
$CIBQ_{IMI}^{\ddagger}$	18.74	155.28	22.16	0.00	0.58
$CIBQ_{total}$	12.82	161.57	28.45	0.00	0.52
$CIBQ_{neonicotinoids}^{*}$	12.73	161.84	28.72	0.00	0.52
$CIBQ_{CLO}$	12.75	163.05	29.93	0.00	0.51
Maximum CLO concentration	9.61	163.16	30.05	0.00	0.49
**Covariate only model (site elevation, bulk density, summer average PPT)**	**11.80**	**167.6**	**34.48**	**0.00**	**0.47**

[†]Chronic invertebrate benchmark quotient (CIBQ) is calculated by dividing the measured concentration for every pesticide detected in a sample by its chronic invertebrate benchmark (usually the No Observable Adverse Effect Concentration (*9*)), summing them for each sample, and taking the maximum value of the 4 samples observed at each site.. [*]$CIBQ_{Neonicotindoids}$ was calculated as for CIBQ except for neonicotinoid compounds. $CIBQ_{IMI}$: Chronic invertebrate benchmark quotient for imidacloprid as is the case with $CIBQ_{CLO}$ except for clothianidin. df: Degrees of freedom. AIC_i: Akaike Information Criterion score for each model. $\Delta_i AIC = (AIC_i - \min(AIC))$. $w_i(AIC) = \exp(-0.5 * \Delta_i AIC)_i / \mathrm{sum}(\Delta_i AIC)$ rounded to 2 digits. Adj-R^2- adjusted coefficient of determination. Bolded model and statistics are the covariate-only model and serves as a baseline by which other models are compared. Covariates in covariate-only model are also included in the other pesticide models. Italic model is the top candidate model.

Disclaimers: Any use of trade, firm, or product names is for descriptive purposes only and does not imply endorsement by the U.S. Government.
